# On the Generic Status of *Asitrichiosoma* Malaise, 1939 (Hymenoptera, Cimbicidae, Cimbicinae) and Its Revision, with Description of Seven New Species †

**DOI:** 10.3390/insects17070745

**Published:** 2026-07-21

**Authors:** Guozhu Deng, Nannan He, Yuchen Yan, Meicai Wei

**Affiliations:** 1 School of Life Sciences and Technology, Central South University of Forestry & Technology, Changsha 410004, China; denggz299@163.com (G.D.); hewera@163.com (N.H.); 2 College of Life Sciences, Jiangxi Normal University, Nanchang 330022, China

**Keywords:** *Asitrichiosoma*, *Trichiosoma*, revision, taxonomy, genetic distance

## Abstract

Due to the conspicuous variation in the present morphology, particularly in the male sex (typically the legs and penis valve), the *Asitrichiosoma* subgenus was established by Malaise, 1939 of the *Trichiosoma* genus. Based on COI data, the genetic distance between *Asitrichiosoma* and *Trichiosoma* is 0.0878, and this genetic distance is significantly different (*p* < 0.001) from the average genetic distance (0.0256) among the species within the genus *Trichiosoma*. Based on morphological and molecular data, we recognize *Asitrichiosoma* as a valid genus. The differences between *Asitrichiosoma* and *Trichiosoma* are discussed in detail. The newly elevated genus is revised, and seven new species are described: *A. bomei* Yan & Wei, sp. nov.; *A. brevicorne* Yan & Wei, sp. nov.; *A. shennong* Yan & Wei, sp. nov.; *A. sinense* Yan & Wei, sp. nov.; *A. omei* Yan & Wei, sp. nov.; *A. poecilomallosum* Yan & Wei, sp. nov.; *A. nigropilosum* Yan & Wei, sp. nov. Descriptions, remarks, illustrations, and a key to the known species of *Asitrichiosoma* are provided. Four new combinations are proposed: *A. himalayanum* (Malaise, 1939), *A. anthracinum* (Forsius, 1930), *A. bombiforme* (Takeuchi, 1939), *A. sikkimense* (Konow, 1897), among which *A. bombiforme* (Takeuchi, 1939), *A. sikkimense* (Konow, 1897) and *A. gansuense* Yan & Wei, 2025 are re-described and illustrated. The distribution of the genus is also briefly discussed.

## Simple Summary

The Cimbicidae represent a relatively small family of herbivorous sawflies, encompassing the largest specimens among true sawflies. Within China, 87 species and 15 genera have been documented thus far. Herein, we utilize an integrative taxonomic approach to elevate the subgenus *Asitrichiosoma* to an independent valid genus and provide detailed descriptions of the generic characteristics, as well as seven new species and four new combinations. The newly recognized genus is reported from East Asia.

## 1. Introduction

True sawflies of the subfamily Cimbicinae are renowned for their morphological diversity in the Cimbicidae [1,2], exhibiting considerable variation in coloration, labrum, mandibles, clypeus, antenna, pubescence, and other morphology features, particularly in male hind legs, which are often inflated and possess spines, as well as in genitalia [3]. This is especially evident in *Trichiosoma*, the second largest genus in the family [2], which was first described by Leach in 1817 and contains approximately 38 species, with its center of diversity in the Palaearctic region [4]. Its type species is *Tenthredo lucorum* Linnaeus, 1758 [=*Trichiosoma lucorum* (Linnaeus, 1758)]. Members of this genus show variation in hair coloration, hind femur, antenna, and penis valve, particularly in males. Konow [5] described *Trichiosoma sikkimense*, and Takeuchi [6] described *T. bombiforme*. These species, including *T. anthracinum* (Forsius, 1930), are easily distinguished from their congeners and can be readily recognized using a key. Due to the conspicuous variation present in male morphology, Malaise [7] established the subgenus *Asitrichiosoma* of genus *Trichiosoma*, describing a new species, *T. himalayanum*, and designating *T. sikkimense* Konow as the type species.

However, there is considerable controversy regarding the taxonomic status of *Trichiosoma* and *Asitrichiosoma*. Malaise [7], Gussakovskij [8], Abe & Smith [9], and Wei et al. [10] recognize *Asitrichiosoma* as a subgenus of *Trichiosoma*, whereas Taeger et al. [4,11] and Vilhelmsen [2] have merged these two genera. Taeger et al. [4] placed the four species in question under *Trichiosoma* and did not recognize the subgeneric status of *Asitrichiosoma*. Furthermore, the monophyly of *Trichiosoma* was not supported by cladistic analyses [2]. Song et al. [12] reported the mitochondrial genome of *T. anthracinum*, and Chen et al. [13] sequenced the mitochondrial genome of *T. vitellinae*. Based on COI data, the phylogenetic position of *T. anthracinum* indicates that it is the sister group of *Trichiosoma* [3,14]. Moreover, the morphological differences between *Asitrichiosoma* and *Trichiosoma* (e.g., head size and shape, body color, body hair, wing macula, and penis valve structure) are distinct and sufficient to justify generic separation. Therefore, we suggest promoting the subgenus *Asitrichiosoma* to a valid genus.

In this paper, we analyze COI gene sequence data from a total of 26 species samples of *Asitrichiosoma* and the genus *Trichiosoma* using maximum likelihood (ML) and Bayesian inference (BI) methods. We construct a phylogenetic tree and conduct genetic distance analysis. In addition, we comparatively study the morphological differences between the two genera and describe seven new species. Four new combinations are proposed: *A. himalayanum* (Malaise, 1939), *A. anthracinum* (Forsius, 1930), *A. bombiforme* (Takeuchi, 1939), and *A. sikkimense* (Konow, 1897). *A. himalayanum* (Malaise, 1939) and *A. sikkimense* (Konow, 1897) are redescribed. Since Yan et al. [1] have already provided the key to Cimbicinae, this paper only provides the key to *Asitrichiosoma*, along with descriptions, remarks, and illustrations.

## 2. Materials and Methods

### 2.1. Morphological Examination and Sample Storage

Specimens were examined with a Leica S8APO stereo microscope. Habitus images were taken with a Nikon D700 digital camera (Nikon, Tokyo, Japan) and a series of images edited using Helicon Focus v 6.7.1 (HeliconSoft, Kharkiv, Ukraine), while detailed images were taken with a Leica Z16 APO/DFC550 (Leica, Wetzlar, Germany). A cylinder of semitransparent plastic was placed around the specimen to disperse the light; that method follows Vilhelmsen [2]. The specimen must be sufficiently relaxed in a moist chamber before dissection. Dissected ovipositor valves, gonoforceps and penis valves were permanently mounted on slides in Arabic gum. Images were produced and composited automatically with a Nikon Ci-L/DS-Fi3 (Nikon, Tokyo, Japan). The series of images produced were focus-stacked using Helicon Focus v 6.7.1 (HeliconSoft, Kharkiv, Ukraine) and further processed with Adobe Photoshop CS 11.0.

The terminology of sawfly genitalia follows Ross [15], and that of general morphology follows Viitasaari [16]. For a few terms (e.g., middle fovea and lateral fovea), we followed Takeuchi [17]. Abbreviations: POL = distance between the mesal edges of the lateral ocelli; OOL = distance between the eye and outer edge of lateral ocelli; OCL = distance between a lateral ocellus and the occipital carina or hind margin of the head.

Types of the new species and most non-type specimens of known species examined in this study are housed in the Asian Sawfly Museum, Nanchang, China (ASMN), with the following exceptions: the holotype and one paratype of *A. sikkimense* and *A. himalayanum* reside in the Berlin Natural History Museum, Germany (BMG); a specimen of *A. sikkimense* is curated at the Natural History Museum, London, UK (BMNH); two paratypes of *A. himalayanum* are kept at the Natural History Museum, Stockholm, Sweden (NHRS); the holotype of *A. anthracinum* belongs to the Zoological Museum of Hamburg (ZMH); the holotype of *A. bombiforme* is preserved in the National Museum of Nature and Science, Tokyo, Japan (NMST); and some specimens of *A. bombiforme* are stored in the Insect Collection of the Forestry College, Northwest Agriculture and Forestry University, Xianyang, China (NAFU).

### 2.2. Phylogenetic and Genetic Distance Analysis

COI was used for phylogenetic analysis (Table 1). *Abia sericea* was used as the out group, and gene sequences were aligned using the MAFFT algorithm v7.313 [18]. Poorly aligned positions in the alignment were removed with Gblocks v. 0.91b within PhyloSuite v. 1.2.3 [19,20]. Phylogenetic analyses were conducted using both Maximum Likelihood (ML) and Bayesian Inference (BI) methods, implemented through plugins in PhyloSuite v. 1.2.3. Specifically, the ML analysis was performed using IQ-TREE v. 2.2.0 with the “ultrafast bootstrap” option, conducting 10,000 bootstrap replicates [21]. For BI analysis, Model Finder v. 2.2.0 was used to select the most appropriate evolutionary model, GTR + F+G4, based on the Bayesian Information Criterion (BIC) [22]. The BI analysis was carried out in MrBayes v. 3.2.7a [23], with four concurrent Markov chains (three cold chains and one heated chain) running for 300,000 generations across two independent runs, sampling every 1000 generations. The first 25% of samples were discarded as burn-in, and convergence was considered to be achieved when the average standard deviation of split frequencies was below 0.01 [24]. The resulting ML and BI trees were visualized using the iTOL website (https://itol.embl.de/, accessed on 12 July 2026) [25].

Estimation of genetic distance was conducted by MAGE 11 [26]. We selected “compute pairwise distances” for comparing these species (Table 1) and used the “bootstrap method” with 1000 bootstrap replicates in the variance estimation method, then analyzed the data by the Kimura-2-parameter model [27]. The genetic distance within species of *Trichiosoma* and the genetic distance between *Asitrichiosoma* and *Trichiosoma* were compared for statistical significance. The results were evaluated using statistical methods, with significance levels indicated as follows: *: *p* < 0.05, **: *p* < 0.01, ***: *p* < 0.001.

**Table 1:** Accession numbers of the GenBank sequences used in phylogenetic analyses.

Species	Accession Number	References
*Agenocimbex maculatus*	OL549450	Unpublished
*Asicimbex* sp.	OM066096	Unpublished
*Cimbex americana*	MG487493	[28]
*Cimbex femoratus*	MZ633015	[29]
*Cimbex connatus*	MZ626015	[29]
*Cimbex luteus*	KC973384	[30]
*Cimbex fagi*	KC972801	[30]
*Labriocimbex sinicus*	MN076591	[14]
*Labriocimbex sinicus*	MN076590	[14]
*Leptocimbex* sp.	KC976797	[30]
*Leptocimbex* sp.	KC976130	[30]
*Leptocimbex* sp.	KC975295	[30]
*Leptocimbex grahami*	MN076599	[14]
*Odontocimbex svenhedini*	OM066098	Unpublished
*Odontocimbex svenhedini*	NC_068104	Unpublished
*Pseudoclavellaria amerinae*	NC_067749	Unpublished
*Pseudoclavellaria amerinae*	OL549456	Unpublished
*Trichiosoma triangulum*	MG487625	[28]
*Trichiosoma lucorum*	MF903226	[28]
*Trichiosoma nanae*	MZ609832	[29]
*Trichiosoma aenescens*	MZ633690	[29]
*Trichiosoma nigricoma*	MZ633482	[29]
*Trichiosoma vitellina*	MN853777	[13]
*Asitrichiosoma anthracinum*	KT921411	[31]
*Asitrichiosoma poecilomallosum*	PZ707350	This study
*Abia sericea*	MZ656415	[29]

## 3. Results

### 3.1. Phylogenetic and Genetic Distance Results

**Figure 1:**
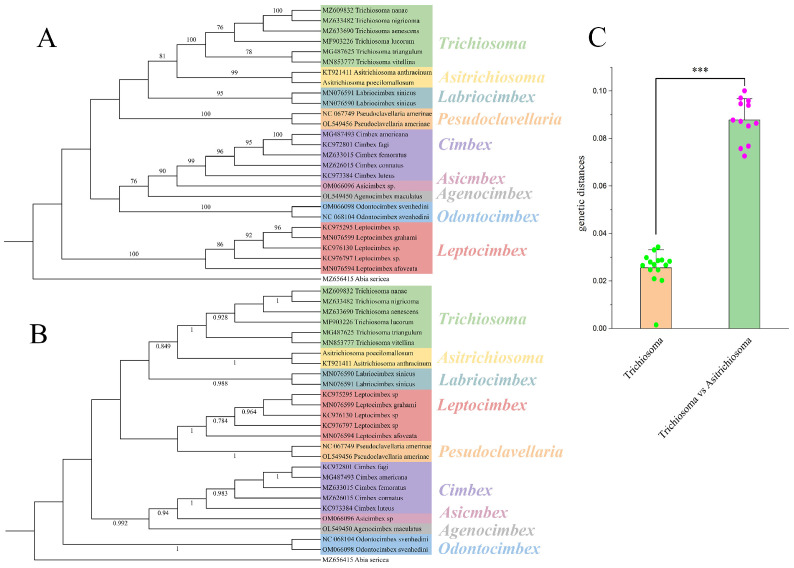
Comparison of phylogenetic trees and genetic distances based on the mitochondrial COI gene. (**A**) Maximum Likelihood (ML) tree. Numbers adjacent to branches represent bootstrap support (values < 75 are omitted). (**B**) Bayesian Inference (BI) tree. Numbers adjacent to branches represent posterior probabilities (values < 0.75 are omitted). (**C**) Comparison of genetic distances within the genus *Trichiosoma* versus between *Asitrichiosoma* and *Trichiosoma*. Statistical significance is indicated as follows: ***: *p* < 0.001. Green and purple dots represent genetic distance.

Phylogenetic trees inferred from Maximum Likelihood (ML) and Bayesian Inference (BI) analyses showed topological incongruence (Figure 1A,B). For instance, the ML tree recovered the topology (((*Trichiosoma* + *Asitrichiosoma*) + *Labriocimbex*) + *Pseudoclavellaria*), whereas the BI tree placed *Pseudoclavellaria* as sister to *Leptocimbex*. However, both ML and BI analyses consistently supported a sister-group relationship between *Asitrichiosoma* and *Trichiosoma*, indicating a close phylogenetic affinity between the genus *Asitrichiosoma* and *Trichiosoma*. Genetic distance analysis revealed an average intraspecific genetic distance of 0.0256 within the genus *Trichiosoma*, whereas the genetic distance between *Asitrichiosoma* and *Trichiosoma* was 0.0878. Further statistical analysis reveals a significant difference (*p* < 0.001) between the genetic distances within *Trichiosoma* and the genetic distance between *Asitrichiosoma* and *Trichiosoma* (Figure 1C), supporting the conclusion that *Asitrichiosoma* does not belong to the *Trichiosoma* genus.

### 3.2. Taxonomy

#### 3.2.1. *Asitrichiosoma Malaise*, 1939

*Asitrichiosoma* Malaise, 1939: 13–16.

**Type species:** *Asitrichiosoma sikkimense* (Konow, 1897)

**Figure 2:**
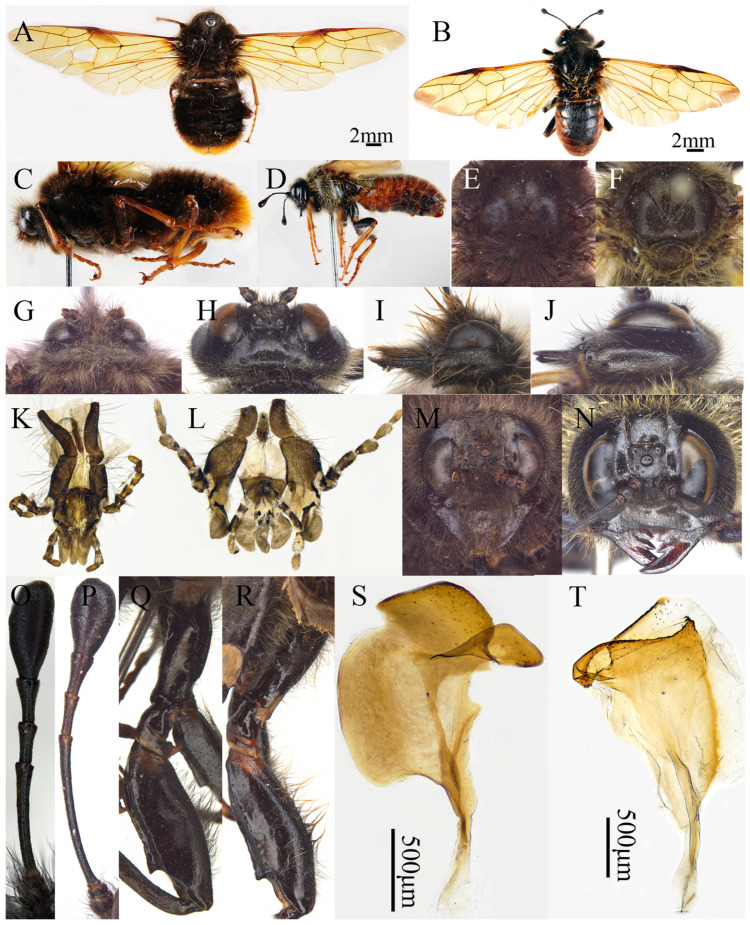
Comparative morphology of difference between the genera *Asitrichiosoma* and *Trichiosoma*. (**A**) Female adult in dorsal view of *A. sikkimense*; (**B**) female adult in dorsal view of *T. vitellinae*; (**C**) female adult in lateral view of *A. sikkimense*; (**D**) female adult in lateral view of *T. vitellinae*; (**E**) thoracic notum of *A. sikkimense*; (**F**) thoracic notum of *T. vitellinae*; (**G**) head in dorsal view of *A. sikkimense*; (**H**) head in dorsal view of *T. vitellinae*; (**I**) head in lateral view of *A. sinensis*; (**J**) head in lateral view of *T. vitellinae*; (**K**) labiomaxillary complex of *A. sinensis*; (**L**) labiomaxillary complex of *T. vitellinae*; (**M**) head in front view of *A. sikkimense*; (**N**) head in front view of *T. vitellinae*; (**O**) antenna of *A. bomei*; (**P**) antenna of *T. vitellinae*; (**Q**) male coxa and femur of *A. sinensis*; (**R**) male coxa and femur of *T. vitellinae*; (**S**) penis valve of *A. sinensis*; (**T**) penis valve of *T. vitellinae*. Scale bars: A–B = 2 mm, S, T = 0.5 mm.

**Figure 3:**
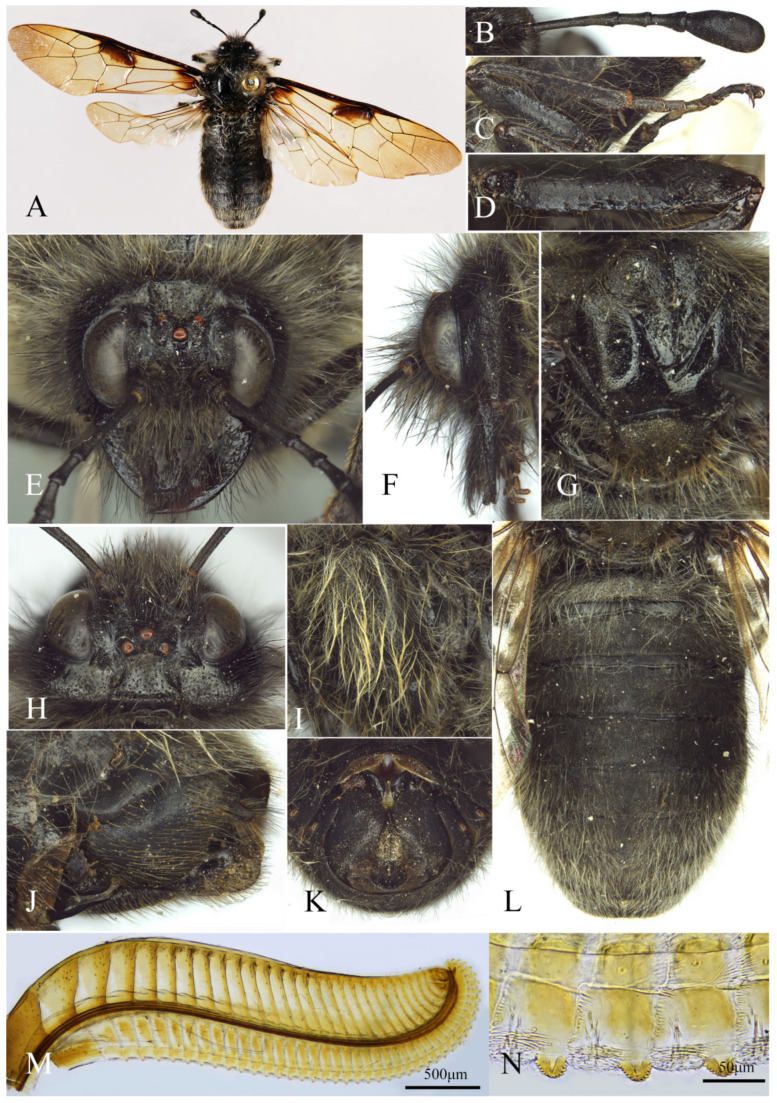
*Asitrichiosoma bomei* Yan & Wei, sp. nov., holotype, female. (**A**) Female adult, dorsal view; (**B**) antenna; (**C**) hind leg; (**D**) hind femur; (**E**) head of female, front view; (**F**) head of female, lateral view; (**G**) mesonotum; (**H**) head of female, dorsal view; (**I**) mesopleuron and metapleuron; (**J**) ovipositor sheath, lateral view; (**K**) ovipositor sheath, ventral view; (**L**) abdominal terga; (**M**) lancet; (**N**) middle serrulae.

**Figure 4:**
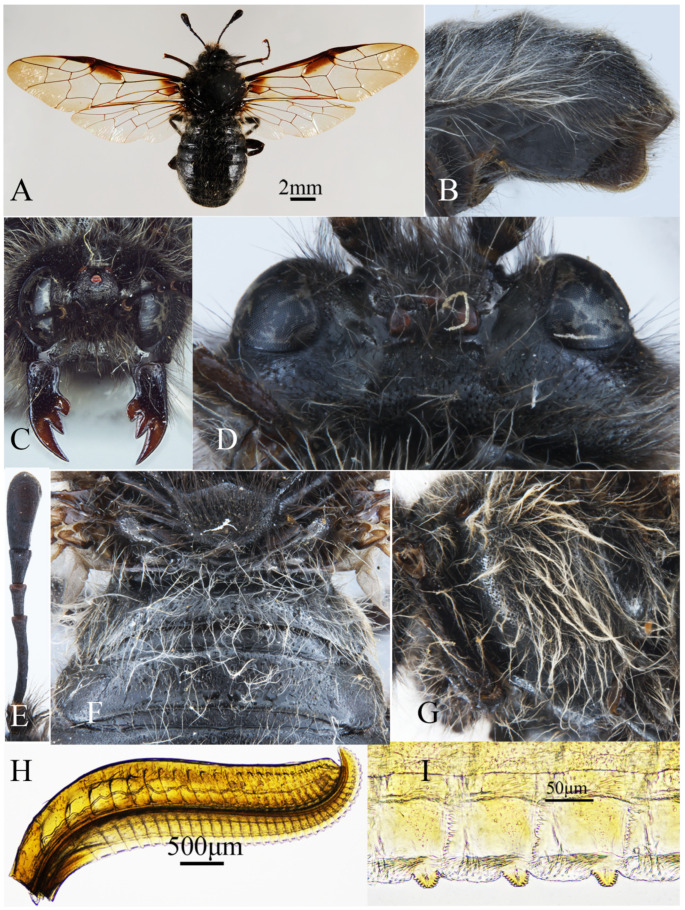
*Asitrichiosoma brevicorne* Yan & Wei, sp. nov., holotype, female. (**A**) Female adult, dorsal view; (**B**) ovipositor sheath, lateral view; (**C**) head in front view; (**D**) head in dorsal view; (**E**) antenna; (**F**) metanotum and base of abdomen; (**G**) mesopleuron and metapleuron; (**H**) lancet; (**I**) middle serrulae.

**Figure 5:**
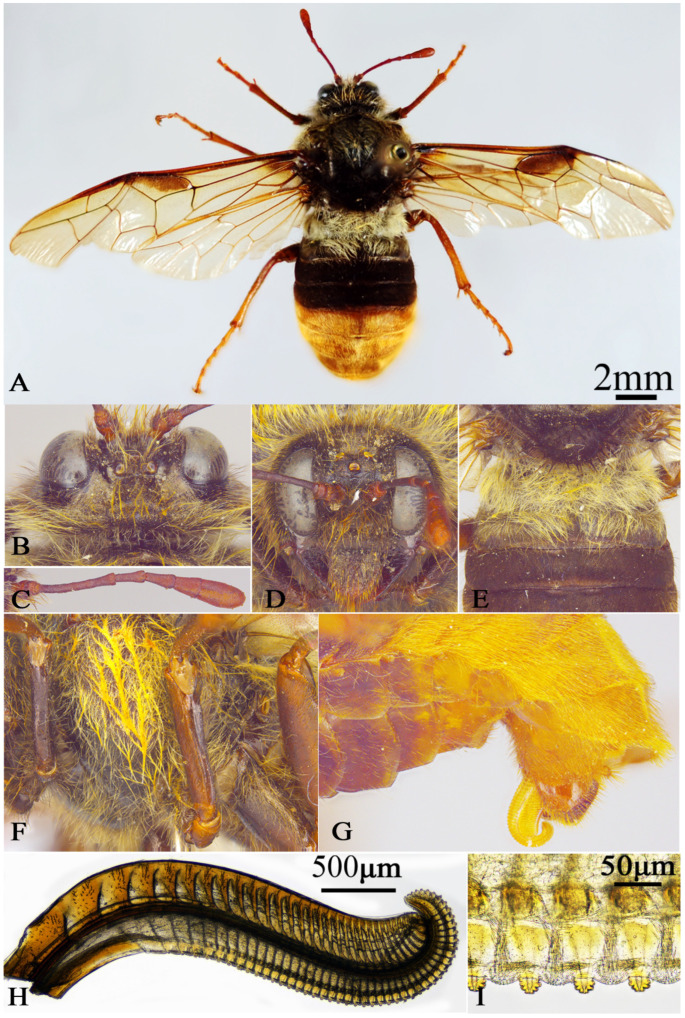
*Asitrichiosoma gansuense* Yan & Wei, 2025, holotype, female. (**A**) Female adult, dorsal view; (**B**) head of female, dorsal view; (**C**) antenna; (**D**) head of female, front view; (**E**) metanotum and base of abdomen; (**F**) mesopleuron and metapleuron; (**G**) ovipositor sheath, lateral view; (**H**) lancet; (**I**) middle serrulae.

**Figure 6:**
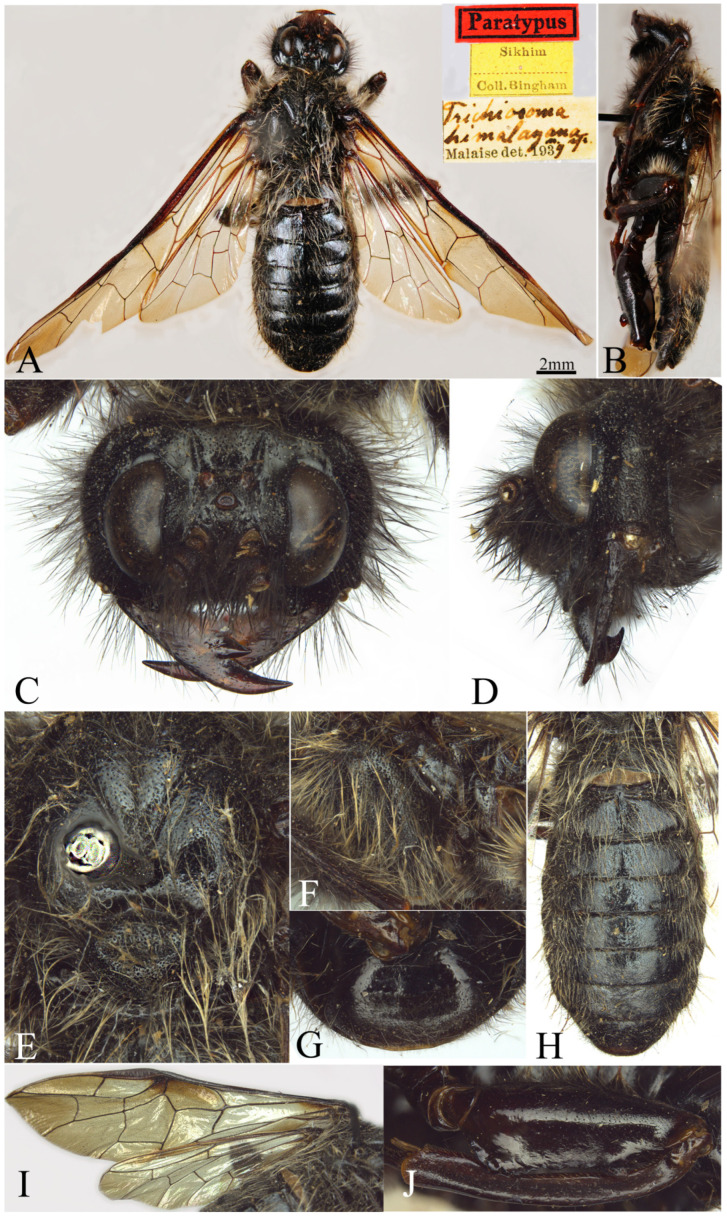
*Asitrichiosoma himalayanum* (Malaise, 1939) comb nov. (**A**) Male adult, dorsal view; (**B**) male adult, lateral view; (**C**) head of male, front view; (**D**) head of male, lateral view; (**E**) mesonotum; (**F**) mesopleuron and metapleuron; (**G**) subgenital plate in ventral view; (**H**) abdominal terga; (**I**) wings; (**J**) hind femur.

**Diagnosis.** This genus is similar to *Trichiosoma* Leach, 1817. *Asitrichiosoma* differs from *Trichiosoma* in the following characters: (1) the body with dense and long hairs, especially head and thorax (Figure 2A,C); (2) the head small, less than 0.75 times as broad as thorax in dorsal view (Figure 2G), the height distinctly more than width in anterior view (Figure 2M), lateral part slightly dilated behind eyes in lateral view (Figure 2I); (3) the antennal club more or less slender and longer than antennomeres 4 and 5 combined, and the club segment longer than its middle width (Figure 2O); (4) the fore wing with a distinct infuscate macula within cell 1M (Figure 2A); (5) the ventral side of the hind femur with two distinct longitudinal carinae, outer carina higher than inner one, especially males with 1 distinct and large denticle near apex (Figure 2Q); (6) the penis valve with a very broad and strongly sclerotized apex lobe, the area between apical lobe and lateral carina with wide groove, wider than lateral carina, strongly sclerotized (Figure 2S).

In *Trichiosoma* Leach 1817, the body hairs sparse (Figure 2B,D); the head large (Figure 2N), more than 0.75 times as broad as thorax in dorsal view (Figure 2H), the lateral part of head strongly dilated behind eyes in dorsal and lateral view (Figure 2J); the antennal club strongly dilated and shorter than antennomeres 4 and 5 combined (Figure 2P); the fore wing without a distinct infuscate macula within cell 1M (Figure 2B); the ventral side of the hind femur only one longitudinal carina (Figure 2R); the penis valve with a narrow and weakly sclerotized dorsal lobe (Figure 2T).

**Description.** Female. Body middle to large-sized, thick and strong; body with dense and long hairs.

**Head.** Head small, less than 0.75 times as broad as thorax in dorsal view, height distinctly more than width in anterior view; clypeus and labrum always black, clypeus distinctly broader than distance between lower margin of eyes, anterior margin with broad and shallow incision; labrum broad and long, weakly narrowed toward base, strongly narrowed toward apex, and its length distinctly longer than width; mandibles flat and elongate, with three teeth, basal one truncate at apex (Figure 4C); maxillary palp with 6 palpomeres, apical 1–2 combined distinctly shorter than palpomere 4; labial palp with 4 palpomeres, short (Figure 2K); malar space about as long as scape and pedicel combined; eyes moderately large, inner margins subparallel, distance between eyes slightly longer than longest axis of eye (Figure 2M); lateral part of head slightly dilated behind eyes in lateral view (Figure 2I) and dorsal view (Figure 2G); postocellar area with median and lateral furrows distinct, frontal carina indistinct (Figure 3H); antennae longer than breadth of head, with 5 antennomeres before club, antennal club more or less slender and longer than antennomeres 4 and 5 combined, club segment longer than middle width (Figure 2O).

**Thorax.** Median furrow of mesoscutal middle lobe normally well developed (Figure 3G); lower margin of mesopleuron without transverse carina (Figure 3I); mesoscutellum flat, anterior margin slightly protruding forward, lateral and posterior margins arc shape; cenchri ovate, metascutellum slightly produced upwards, without spines (Figure 3G); fore wing with a distinct infuscate macula within cell 1M, basal 1/3 of anal cell with a short crossvein, forming two closed cells A (Figure 3A); ventral side of hind femur with two distinct longitudinal carinae, outer carina higher than inner one, with 1 distinct denticle near apex, hind tibia spurs blunt (Figure 10I).

**Figure 7:**
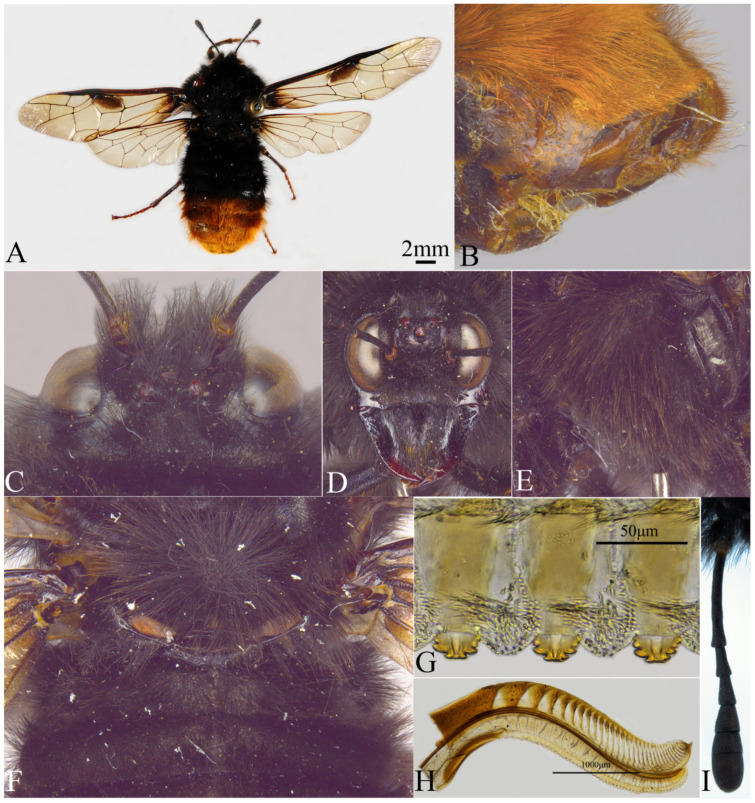
*Asitrichiosoma nigropilosum* Yan & Wei, sp. nov. (**A**) Female adult, dorsal view; (**B**) ovipositor sheath, lateral view; (**C**) head of female, dorsal view; (**D**) head of female, front view; (**E**) mesopleuron and metapleuron; (**F**) metanotum and base of abdomen; (**G**) middle serrulae; (**H**) lancet; (**I**) antenna.

**Abdomen.** Abdominal terga with dense long hairs; ovipositor sheath as long as middle tibia, apex slightly or not protruded beyond end of abdomen (Figure 3J); lance long, usually weakly broadened beyond apex, apical incision broad and a hook distinct (Figure 7H); lancet narrow and long, weakly tapering toward apex with 37–51 serrulae, serrulae small and remote to each other, slightly protruding beyond apex of cypsella, annular spines very long and dense (Figure 3N), cypsella densely pilose (Figure 3M).

**Figure 8:**
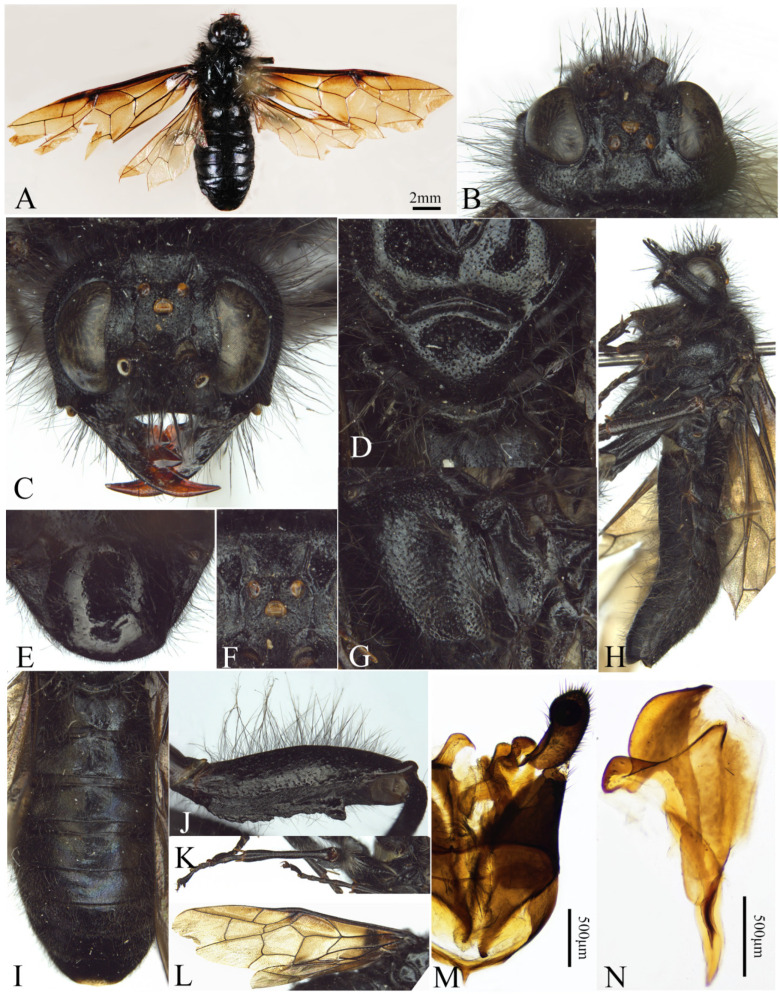
*Asitrichiosoma omei* Yan & Wei, sp. nov. (**A**) Male adult, dorsal view; (**B**) head of male, dorsal view; (**C**) head of male, front view; (**D**) scutellum; (**E**) subgenital plate in ventral view; (**F**) ocellar area; (**G**) mesopleuron and metapleuron; (**H**) adult male, lateral view; (**I**) abdominal terga; (**J**) hind femur; (**K**) tibia and tarsus; (**L**) wings; (**M**) gonoforceps; (**N**) penis valve.

**Figure 9:**
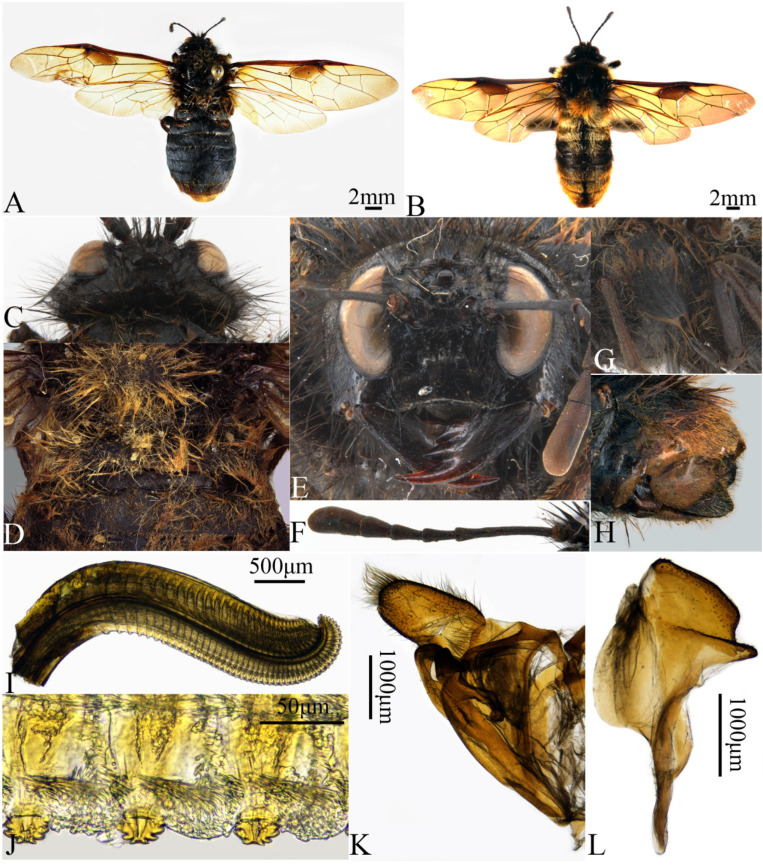
*Asitrichiosoma poecilomallosum* Yan & Wei, sp. nov. Holotype. (**A**) Female adult, dorsal view; (**B**) male adult, dorsal view; (**C**) head of female, dorsal view; (**D**) metanotum and base of abdomen; (**E**) head of female, front view; (**F**) antenna; (**G**) mesopleuron and metapleuron; (**H**) ovipositor sheath, lateral view; (**I**) lancet; (**J**) middle serrulae; (**K**) gonoforceps; (**L**) penis valve.

**Male.** Structure similar to female except for following parts: anterior incision of clypeus more clear than female, labrum wider than female (Figure 13A); abdominal terga with distinct median longitudinal ridge (Figures 6H, 8I, 11B and 13H); subgenital plate prominent, anterior margin rounded, arc-shaped posterior margin, slightly longer than broad (Figures 6G, 8E and 13G); middle and hind coxae and femora distinctly elongated, coxae ventrally with 2 long denticles (Figures 6B and 13I), femora ventrally with two distinct longitudinal carinae, outer carina more elevated than inner, apex with 1 large denticle near apex (Figures 6I, 8J and 13E); penis valve with a very broad and strongly sclerotized apex lobe, the area between apical lobe and lateral carina with wide groove, wider than lateral carina, strongly sclerotized (Figures 8N, 9L and 12R).

#### 3.2.2. Key to the Species of the Genus *Asitrichiosoma* from China

1. Posterior of abdomen of both sexes without reddish brown hairs; body hairs long but not very dense; body length less than 16 mm in females, less than 20 mm in males ………………………………………………………………………………………...2

- Posterior of abdomen of both sexes’ hairs reddish brown; body hairs of both sexes very dense, especially on thorax; fore wing of males largely hyaline; body length of females 16.5–20 mm, males not shorter than 23 mm …………………………………………………………………………………………..8

2. Abdominal terga densely microsculptured in both sexes, abdominal terga 2–8 not distinctly shiny; fore and hind wings of males dark smoky brown ………………….3

- Abdominal terga of both sexes with weak microsculptures, abdominal terga 2–8 distinctly shiny; fore and hind wings of males largely hyaline, infuscate macula clear; femora and abdominal terga all black, tibiae blackish brown ……………………………………………………………………………………….6

3. Antenna shorter, 1.2× longer than head breadth, antennomere 3 shorter than or equal to longest axis of eye; body hairs grayish brown; femora, tibiae and abdominal terga 7 and 8 black ……………………………………………………………………………..…4

- Antenna longer, 1.5× longer than head breadth, antennomere 3 distinctly longer than longest axis of eye, 1.7× antennomeres 4 and 5 combined; female body hairs almost yellowish brown; vein 2m-cu in fore wing joining to 1r-m vein apical or located on inner side …………………………………………………………………………………..5

4. Postocellar area flat, without postocellar furrow; lateral furrow distinctly curved, strongly divergent backwards; frontal area without regular oblique ridges, lateral furrow with fine microsculpture, bottom not smooth; upper edge of antennal toruli distinctly upturned; antennal club completely fusion, 3rd antennomere 1.4× antennomeres 4 and 5 combined; middle of median mesoscutal groove deeper than two terminals, middle of notaulus shallower than two terminals; fore wing vein 2m-cu joining cell 2Rs inner of vein 1r-m, vein 2r joining inner side of middle of cell 2Rs. Tibet ……………………………………………….…………….. ***A. bomei*** Yan & Wei, sp. nov.

- Posterior of postocellar area elevated, with distinct postocellar furrow; lateral furrow not curved, uniformly and weakly divergent backward; frontal area with regular oblique ridges, bottom distinctly smooth, upper edge of antennal toruli not distinctly upturned; inner side of antennal club segmented, antennomere 3 about 1.2× antennomeres 4 and 5 combined; median mesoscutal groove and notaulus consistent depth at middle and two terminals; fore wing vein 2m-cu joining cell 2Rs outer side of vein 1r-m, vein 2r joining middle of cell 2Rs. Sichuan ……………………………………………..… ***A. brevicorne*** Yan & Wei, sp. nov.

5. Coxae and tibiae black; posterior of abdominal terga entirely black. Male unknown. Hubei …………………………………………..…….. ***A. shennong*** Yan & Wei, sp. nov.

- Ventral side of femora and tibiae light brown; posterior of abdominal terga part light brown. Sichuan ………………………………………… ***A. sinense*** Yan & Wei, sp. nov.

6. Fore wing without anal crossvein; abdominal terga sparsely or indistinctly punctured; thorax and abdomen with short, dark hairs; antenna with 7 antennomeres, antennomeres 6 and 7 clearly separated. ………………………………………………....7

- Anal crossvein in fore wing distinct; abdominal terga densely punctured; thorax and abdomen with long, pale hairs; antenna with 6 antennomeres, antennomeres 6 and 7 indistinctly separated. Himalayan region ……………***A. himalayanum*** (Malaise, 1939)

7. Middle of abdominal terga almost naked, with very sparse and short hairs; apex lobe groove of penis valves short, anterior margin of upper part almost vertical, height about 1/2 the width of sclerotized dorsal margin, posterior angle of apex lobe located back side of valvispina, protruding angle of paravalva located in upper 1/3 of apex lobe. Northeastern, Qinghai……………………….. ***A. anthracinum*** (Forsius, 1930)

- Middle of abdominal terga with fine hair; apex lobe groove of penis valves broad, anterior margin arcuate, height more than width of sclerotized dorsal margin, posterior angle of apex lobe located front side of valvispina, protruding angle of paravalva located in middle of apex lobe. Sichuan ………………. ***A. omei*** Yan & Wei, sp. nov.

8. Body entirely black, with bronzy luster especially on mesonotum; legs black, with tibiae and tarsus yellowish brown, and femora more or less bluish; body hairs as in bumble bee, head largely blackish except the supraclypeal area, vertex and posterior margin with longer and yellow hairs; scutellum and entire terga with dense, fulvous cottony hairs, abdominal terga 1–3 yellow, tergum 4 black, terga 5–8 reddish brown hairs. Gansu, Shaanxi, Japan…………………………. ***A. bombiforme*** (Takeuchi, 1939)

- At least 2 segments of posterior of abdominal terga reddish brown both sexes …………………………………………………………………………………………9

9. Abdominal terga 5–8 all reddish brown; fore wing entirely hyaline except for smoky macula on base and cell 1M; hind trochanters to tarsus yellowish brown. Gansu ……………………………...…………………….. ***A. gansuense*** Yan & Wei, 2025

- Posterior of abdominal terga only 1–2 segments reddish brown; fore wing distinctly smoke brown apically or entirely smoky brown; at least dorsal side of hind femur black ……………………………...……………………………………...…………………10

10. Female head, thorax and abdominal terga 1–4 entirely with black or dark-brown hairs; antenna short, about 1.2× breadth of head ……………………………………...……...11

- Median mesoscutal lobe, mesoscutellum and pleuron with yellowish brown hairs, head, lateral mesoscutal lobe, both sides of abdominal tergum 3 and largely of tergum 4 with black hairs, other abdominal terga with yellowish brown hairs; antenna relatively long, 1.4× head breadth. Hubei, Hunan, Chongqing, Zhejiang, Sichuang……………………………….……… ***A. poecilomallosum*** Yan & Wei, sp. nov.

11. Posterior four segments of abdominal terga with reddish brown hairs, rest of body black; below of pterostigma smoky macula in fore wing covering largely cell 1M; anterior half part of median mesoscutal groove extremely broad; vein 2m-cu straight, vein cu-a directly joining vein 1M at apex; abdominal tergum 1 densely microsculptured, dull; femora and tibiae black in female. Henan, Shaanxi..…………………………………………… …***A. nigropilosum*** Yan & Wei, sp. nov.

- Posterior two segments of abdominal terga with reddish brown hairs, rest of body largely with dark-brown hairs in female, thorax and abdomen largely with light-brown hairs in male; below of pterostigma smoky macula in fore wing covering the upper half of cell 1M; median mesoscutal groove normal and not widened; vein 2m-cu distinctly curved, vein cu-a directly joining vein 1M at apex; abdominal tergum 1 weakly microsculptured, with noticeably glossy surface; femora and tibiae not entirely black. Himalayan region …….……………………***A. sikkimense*** (Konow, 1897)

#### 3.2.3. *Asitrichiosoma bomei* Yan & Wei, sp. nov.

urn:lsid:zoobank.org:act:B73AEC4E-D2C9-43F4-80FB-C58FE13E13A1

(Figure 3A–N)

**Material examined. *Holotype*** female, CHINA: Tibet, Bomi County, Zhamu Highway, 0.9K, 3039 m, 29°50.933′ N, 95°43.467′ E, 18 July 2014, leg. Jun Xu, Mei Qin (ASMN).

**Diagnosis.** The species is similar to *A. brevicorne* Yan & Wei, sp. nov., but it differs from the latter in the following characters: postocellar area flat, without postocellar furrow (Figure 3H); lateral furrow distinctly curved, strongly divergent backwards; frontal area without regular oblique ridges, lateral furrow with fine microsculpture, bottom not smooth (Figure 3E); upper edge of antennal toruli distinctly upturned; antenna with 6 antennomeres, antennal club completely fused, 3rd antennomere 1.4× antennomeres 4 and 5 combined (Figure 3B); middle of median mesoscutal groove deeper than two terminals, middle of notaulus shallower than two terminals (Figure 3G); fore wing vein 2m-cu joining cell 2Rs inner side of vein 1r-m, vein 2r joining inner side of middle of cell 2Rs (Figure 3A).

**Coloration.** Body black; apical 1/2 of mandibles blackish brown; wings brown, veins largely blackish brown, basal of fore wing hyaline, pterostigma black, infuscate macula covering basal of fore wing and upper part 2/3 of cell 1M, hind wing hyaline, veins Sc + R and M yellowish brown (Figure 3A); head hairs largely blackish brown, vertex mixed long hairs blackish brown at base and yellowish brown at apex, mesonotum hairs yellowish brown, pleuron, abdominal terga 3–8, coxae and femora hairs yellowish white (Figure 3L); abdominal terga 1–2 hairs grayish white, tibiae with reddish brown setae (Figure 3C), sheath with yellowish brown hairs.

**Sculptures.** Body finely and densely microsculptured; postocular area very distinctly microsculptured; below of antenna with broad and shallow punctures, interspaces between punctures smooth and shiny; median and lateral mesoscutal lobes with coarse and broad punctures, interspaces between punctures wide, smooth and shiny, scutellum and mesopleuron densely and deeply punctured; abdominal tergum 1 very densely punctured, weakly microsculptured, other terga without punctures, finely and densely microsculptured; abdominal sternum, middle and hind coxae and femora densely microsculptured, luster weak.

**Head.** Clypeus thickened in middle; malar space 1.4× diameter of middle ocellus; distance between inner margins of eyes as long as longest axis of eye; frontal area subround, frontal ridge low and obtuse, without regular oblique ridges (Figure 3E); POL: OOL: OCL = 3: 3.3: 5.2 (Figure 3H); postocellar area flat, 1.5× broader than long, without postocellar furrow; lateral furrow distinctly curved, strongly divergent backwards; upper edge of antennal toruli distinctly upturned, antenna with 6 antennomeres, 1.3× head breadth, antennomere 3 about 1.4× antennomeres 4 and 5 combined, antennal club not segmented, 1.2× antennomeres 4 and 5 combined (Figure 3B); hairs on vertex of head dense and long.

**Thorax.** Middle of median mesoscutal groove deeper than two terminals, middle of notaulus shallower than two terminals; transverse furrow deep in anterior of mesoscutellum, anterior margin of mesoscutellum broad and obtuse, posterior margin round; posterior corner of metascutellum obtusely convex; cenchrus narrow and long, distance between cenchri 1.6× longest axis of a cenchrus; pronotum and mesonotum with sparse long hairs, mesopleuron and scutellum with dense and long hairs; fore wing vein 2m-cu joining cell 2Rs inner side of vein 1r-m, distance about 1/2 length of 1r-m (Figure 3A).

**Abdomen.** Abdomen hairs sparse and long largely, terga 6–8 hairs dense (Figure 3L). Lancet with 42 serrulae (Figure 3M), middle serrulae, membranous area between serrulae flat, distance between serrulae about 1.6× basal breadth of a serrula, middle serrulae protruding, subbasal teeth small, each side with about 6 min subbasal teeth (Figure 3N).

**Distribution:** China (Tibet).

**Etymology:** The species name is derived from its type locality, Bomi.

#### 3.2.4. *Asitrichiosoma brevicorne* Yan & Wei, sp. nov.

urn:lsid:zoobank.org:act:B41B8177-16FD-4DB6-AB40-9B23A6860651

(Figure 4A–I)

**Material examined. *Holotype*** female, CHINA: Sichuan Province, Kangding County, 17 May 1986; leg. T. Naito (ASMN).

**Diagnosis.** The species is similar to *A. bomei* Yan & Wei, sp. nov., but it differs from the latter in the following characters: posterior of postocellar area elevated, with distinct postocellar furrow (Figure 4D); lateral furrow not curved, uniformly and weakly divergent backward; frontal area with regular oblique ridges, bottom distinctly smooth, upper edge of antennal toruli not distinctly upturned (Figure 4C); antenna with 7 antennomeres, short and 1.2× head breadth, antennal club segmented, antennomere 3 about 1.2× antennomeres 4 and 5 combined (Figure 4E); median mesoscutal groove and notaulus of consistent depth at middle and two terminals; fore wing vein 2m-cu joining cell 2Rs outer side of vein 1r-m, vein 2r joining middle of cell 2Rs (Figure 4A).

**Description.** Holotype, female. Body length 15 mm (Figure 4A).

**Coloration.** Body black (Figure 4A); wings yellow and hyaline, veins largely blackish brown, pterostigma black, infuscate macula covering basal of fore wing and upper part 1/2 of cell 1M (Figure 4A); tarsus and tibiae of legs blackish brown; vertex of head hairs largely grayish brown, mixed with long black hairs, postocular area with grayish white hairs; notum hairs blackish brown, pleuron hairs yellowish brown; sides of abdominal terga hairs grayish white (Figure 4B), coxae and femora hairs yellowish white.

**Sculptures.** Body largely with dense microsculptures; apical 1/2 of mandibles smooth, strongly shiny, basal 1/2 of labrum and mandibles with fine and dense punctures, interspaces smooth, bottom of frontal area distinctly smooth (Figure 4C); mesonotum with minute and shallow punctures, interspaces smooth, shiny, mesopleuron deeply and densely punctured; abdomen tergum 1 densely punctured, weakly microsculptured, other terga without punctures, with dense microsculpture; middle and hind coxae and femora with sparse, broad punctures, interspaces smooth and shiny.

**Head.** Labrum thickened in middle; mandibles thickened at base; malar space 1.5× diameter of middle ocellus, distance between inner margins of eyes as long as longest axis of eye; frontal area trapezoidal, with regular oblique ridges (Figure 4C); POL: OOL: OCL = 2: 3.3: 4.6 (Figure 4D); Posterior of postocellar area elevated, with distinct postocellar furrow; lateral furrow not curved, uniformly and weakly divergent backward, 1.7× broader than long; upper edge of antennal toruli not distinctly upturned; antenna with 7 antennomeres, short and 1.2× head breadth; antennomere 3 clearly shorter than longest axis of eye, 1.2× antennomeres 4 and 5 combined, antennal club segmented, 1.1× antennomeres 4 and 5 combined (Figure 4E); Long hairs on gena clearly longer than 1/3 head width in dorsal view, vertex and behind eyes of head with long and dense hairs.

**Thorax.** Median mesoscutal groove and notaulus of consistent depth at middle and two terminals; anterior margin of mesoscutellum blunt triangle; metascutellum slightly protruding backwards, without spines; cenchri oblong, distance between cenchri 1.5× longest axis of a cenchrus; pronotum and mesonotum with sparse and long hairs, mesopleuron with dense and long hairs, scutellum and metanotum with slightly sparse hairs; fore wing vein 2m-cu joining cell 2Rs outer side of vein 1r-m, distance about 1/4 length of 1r-m,vein 2r joining middle of cell 2Rs (Figure 4A).

**Abdomen.** Abdomen with sparse and long hairs (Figure 4B), lateral margins of terga 7–8 with dense and long hairs (Figure 4F); Lancet with 37 serrulae (Figure 4H), membranous area between serrulae slightly protruding, distance between serrulae about 2.4× basal breadth of a serrula, middle serrulae protruding, subbasal teeth small, each side with about 7 min subbasal teeth (Figure 4I).

**Distribution:** China (Sichuan).

**Etymology:** The species epithet is derived from the Latin “brevi” and “corne,” referring to the short antenna.

#### 3.2.5. *Asitrichiosoma gansuense* Yan & Wei, 2025.

*Asitrichiosoma gansuense* Yan & Wei, 2025, vol, 3, 56 [32].

(Figure 5A–I)

**Material examined. *Holotype*** female, CHINA: Gansu Province, Mt. Xiaolong, Dangchuan Forest Farm, 1700m, 34°24.413′ N 106°08.032′ E, 1 June 2009, leg. Fan Hui (ASMN).

**Paratype:** CHINA: Gansu Province, Gansu Forestry Research Institute, Experimental Forest Farm, 1600 m, 34°20.286′ N 106°00.591′ E, 6 May 2010, leg. Xin Heng (ASMN).

**Diagnosis:** The species is similar to *A. poecilomallosum* Yan & Wei sp. nov., but it differs from the latter in the following characters: apical abdomen terga 1–4 entirely reddish brown (Figure 5G); fore wing entirely hyaline except for smoky macula on base and cell 1M (Figure 5A); hind trochanter and tarsus entirely yellowish brown (Figure 5A).

**Description.** Female. Body length 17 mm, thick and strong (Figure 5A).

**Coloration.** Body largely black; apical of mandibles and labrum reddish brown, clypeus black–brown, antenna largely reddish brown (Figure 5D); pronotum with macula reddish brown, cenchri dark blackish brown; abdominal terga 5–8 reddish brown, sterna 1–4 dark brown, sterna 5–10, sheath reddish brown; wings hyaline pale yellowish brown, infuscate macula covering upper 2/3 of cell 1M and base of fore wing, pterostigma blackish brown, veins largely brown, vein C reddish brown (Figure 5A); coxae, trochanters and fore femur dark reddish brown, middle and hind femora reddish brown, outer side with black stripe macula, tibiae and tarsus reddish brown; body with multicolored hairs in dorsal view, head largely yellowish brown hairs, densely, both sides of clypeus and labrum mixed blackish brown hairs, pronotum, anterior part of mesonotum, mesopleuron with yellowish brown hairs, posterior part of mesonotum and scutellum with blackish brown hairs, metanotum and abdominal terga 1–2 with yellowish white hairs, terga 3–4 with black hairs, terga 5–8 and sheath with reddish brown hairs.

**Sculptures.** Head densely microsculptured without punctures, weakly shiny; meso-notum and pleuron weakly punctured, smooth interspaces, slight shiny; abdominal sterna and legs strongly shiny, hind coxa with oily sheen, abdominal terga not shiny.

**Head.** Labrum elongate, anterior margin of clypeus indistinctly concave, malar space 1.4× diameter of middle ocellus; distance between inner margins of eyes 0.9× longest axis of eye; frontal furrow deeply impressed; frons area circular, frontal ridge low and blunt (Figure 5D); POL: OOL: OCL = 2: 2.6: 4.5; postocellar furrow distinct, postocellar area clearly longer than wide, lateral furrow deep, slightly divergent (Figure 5B); postocular area slightly enlarged; antenna with 7 antennomeres, 1.4× the head breadth, antennomere 3 about 1.3× the antennomeres 4 and 5 combined, club 1.3× the antennomeres 4 and 5 combined, widest 2× apical breadth of antennomere 3 (Figure 5C).

**Thorax.** Mesopleuron with a weak transverse carina; distance between cenchri 2.6× longest axis of a cenchrus; metascutellum bluntly protruding; thorax with dense and long hairs, hairs on median mesoscutal lobe sparser than mesopleuron; vein 2m-cu joining cell 2Rs on inner side of vein 1r-m (Figure 5A).

**Abdomen.** Abdominal terga 1 and 2 with dense, long hairs, remaining abdominal terga with slightly sparse, short hairs (Figure 5E); Lancet with 51 serrulae (Figure 5H), middle serrulae as Figure 5I, annular spine dense, membranous area between serrulae slightly protruding, distance between serrulae about 1.8× basal breadth of a serrula (Figure 5I); middle serrulae protruding, subbasal teeth slightly large, each side with about 4 min subbasal teeth.

**Distribution:** China (Gansu).

**Etymology:** The species epithet is derived from the type locality.

#### 3.2.6. *Asitrichiosoma himalayanum* (Malaise, 1939) comb. nov.

*Trichiosoma* (*Asitrichiosoma*) *himalayanum* Malaise, 1939: 14.

urn:lsid:zoobank.org:act:6A0463BB-F42F-4B21-B45F-FBCC4D9E9EB5

(Figure 6A–J)

**Material examined.** Male, CHINA: Bingham; Sikhim Coll. (BMG)

**Diagnosis.** The species is similar to *A. anthracinum* (Forsius, 1930), but it differs from the latter in the following characters: anal crossvein in fore wing distinct (Figure 6I); abdominal terga with slightly dense punctures; thorax and abdomen with pale, long hairs; antenna 6 segments, antennomeres 6 and 7 with indistinct segmentation.

**Description.** Male. Body length 21.5 mm, body black (Figure 6A).

**Coloration.** Apical 1/2 of mandibles blackish brown, ocellus dark reddish brown, cenchri blackish brown (Figure 6C,L); tibiae and tarsus blackish brown (Figure 6J); wings pale brown and hyaline, fore wing veins black, pterostigma black, infuscate macula covering 1/2 cell 1M and base of cell 1Rs, hind wing veins largely blackish brown, apical angles of fore and hind wings with smoky macula (Figure 6I); head hairs largely blackish brown, mixed long black hairs (Figure 6C); thorax hairs yellowish brown (Figure 6E); abdominal terga hairs yellowish white (Figure 6H); coxae and femora with yellowish brown hairs.

**Sculptures.** Body finely and densely punctured, microsculpture weak; clypeus and mandibles broadly and distinctly punctured, frontal area distinctly microsculptured (Figure 6C); mesonotum finely and densely punctured, mesopleuron and sternum deeply and densely punctured; abdominal terga distinctly microsculptured on median carina, other with slightly weak microsculpture and dense punctures, feebly shiny; abdominal sterna, middle and hind coxae and femora with sparse punctures, interspaces smooth and shiny.

**Head.** Long hairs on gena, vertex and behind eyes of head clearly densely (Figure 6C); malar space 1.8× diameter of middle ocellus, distance between inner margins of eyes 0.9× longest axis of eye; frontal area circular, front ridge low, curved in middle (Figure 6C); POL: OOL: OCL = 2.4: 3.1: 5.1; postocellar furrow weak, middle furrow broad and shallow (Figure 6C); postocellar area 1.2× broader than long, lateral furrow fine and deep, slightly divergent backwards; antenna with 6 antennomeres, club with indistinct segmentation.

**Thorax.** Median mesoscutal groove fine and weak, notaulus distinct (Figure 6E); anterior margin of mesoscutellum arcuate, with a longitudinal groove on the top; mesoscutellum slightly protruding upwards, without spines; distance between cenchri 2.6× longest axis of a cenchrus; pleuron and scutellum hairs dense and long; veins 1r-m and 2m-cu in fore wing completely joining at apex, basal 1/3 of anal cell with distinct crossvein (Figure 6I).

**Abdomen.** Abdominal tergum 1 with dense hairs, middle of other abdominal terga with sparse hairs, lateral margins densely (Figure 6H).

**Distribution:** China (Himalayan region).

#### 3.2.7. *Asitrichiosoma nigropilosum* Yan & Wei, sp. nov.

urn:lsid:zoobank.org:act:EDC637D5-23C1-4CB6-8DC9-6D9CC2553062

(Figure 7A–I)

**Material examined. *Holotype*** female, CHINA-Henan Province, Lushi County, Mt. Yuhuang, Bendangou, 1447 m, 33°45.200′ N, 110°50.210′ E, 1 May 2019, leg. Shuxin Liu and Yiwen Zhang (ASMN).

**Diagnosis:** The species is similar to *A. sikkimense* (Konow, 1897), but it differs from the latter in the following characters: posterior four segments of abdominal terga with reddish brown hairs, rest of body black (Figure 7A); below of pterostigma smoky macula in fore wing covering largely cell 1M (Figure 7A); anterior half part of median mesoscutal groove extremely wide; vein 2m-cu straight, vein cu-a not directly joining to vein 1M at apex (Figure 7A); abdominal tergum 1 densely microsculptured, dull; femora and tibiae black in female.

**Description.** Female. Body length 20 mm, body black, thick and strong (Figure 7A).

**Coloration.** Mandibles apically and ocellus reddish brown (Figure 7D); cenchri yellowish brown; abdominal terga 7–8 dark reddish brown, sterna 6–10 blackish brown, sheath reddish brown; wings pale yellowish brown hyaline, darkly infuscate macula on base of fore wing and largely cell 1M, pterostigma black, wings veins largely black, veins C, Sc + R and J yellowish brown (Figure 7A); legs black, tarsus reddish brown; body with dense and long hairs, except for reddish brown hairs on abdominal terga 5–8; sheath margin densely with reddish brown short hairs, remaining body hairs all black.

**Sculptures.** Body finely and densely punctured; head and thorax not obviously shiny, dorsal and mesopleuron with smooth interspaces between punctures, shiny; abdominal tergum 1 with dense microsculpture, without luster, abdominal terga dim, abdominal sterna slightly shining; coxae with oily sheen.

**Head.** Clypeus 1.6× distance between lower margins of eyes, anterior margin with broad, arc-shaped depression, malar space 1.5× diameter of middle ocellus; distance between inner margins of eyes as long as longest axis of eye; frontal area circular, frontal ridge low and blunt (Figure 7D); postocellar furrow indistinct; POL: OOL: OCL = 3: 3.2: 4.2; postocellar area clearly longer than wide, lateral furrow deep, divergent backwards (Figure 7C); postocular area slightly enlarged; antenna with 8 antennomeres, 1.2× head breadth, antennomere 3 about 1.5× antennomeres 4 and 5 combined, club 1.5× antennomeres 4 and 5 combined, antennomere 8 distinctly segmented marks (Figure 7I).

**Thorax.** Median mesoscutal groove extremely broad in anterior half; mesopleuron with ventral margin without transverse carinae; mesoscutellum flat, anterior margin slightly arcuate; cenchri ovate, distance between cenchri 1.7× longest axis of a cenchrus; metascutellum straight, not protruding; pronotum and mesepisternum with dense and long hairs; median and lateral mesoscutal lobe with slightly sparse hairs, mesoscutellum, metanotum and metapleuron with dense hairs; veins 2m-cu and 1r-m in fore wing completely joining at apex, vein 2m-cu straight, vein cu-a not directly joining to vein 1M at apex, dorsal side of middle and hind femora with dense, long hairs, forming a brush-like structure.

**Abdomen.** Abdominal terga 1–4 with a median longitudinal ridge, median longitudinal ridge and lateral margins with dense, long hairs, remaining areas with dense, short hairs; Lancet with 50 serrulae, annular spine dense, membranous area between serrulae protruding, distance between serrulae about 1.8× basal breadth of a serrula (Figure 7H); middle serrulae slightly protruding, subbasal teeth large, each side with about 3 min subbasal teeth (Figure 7G).

**Distribution:** China (Henan, Shaanxi).

**Etymology:** The new species name is derived from the Latin words “nigro” and “pilosum”, referring to the body dense hairs, predominantly black.

#### 3.2.8. *Asitrichiosoma omei* Yan & Wei, sp. nov.

urn:lsid:zoobank.org:act:87A2DDE7-3A39-4A18-B899-EF53ADDADD15

(Figure 8A–N)

**Material examined. *Holotype*** male, CHINA: Sichuan Province, Mt. Emei, Gold Peak; 3067 m, 29°31.369′ N, 130°20.188′ E, 3 July 2006; leg. Yihai Zhong (ASMN).

**Diagnosis.** The species is similar to *A. anthracinum* (Forsius, 1930), but it differs from the latter in the following characters: Middle of abdominal terga with fine hair, not naked (Figure 8I); apex lobe groove of penis valves broad, anterior margin arcuate, height more than width of sclerotized dorsal margin, posterior angle of apex lobe located front side of valvispina, protruding angle of paravalva located in middle of apex lobe (Figure 8N).

**Description.** Male. Body length 16 mm, body and legs black (Figure 8H).

**Coloration.** Apical 1/2 of mandibles and ocellus reddish brown (Figure 8C); wings dark smoky brown and largely hyaline, infuscate macula covering 1/3 cell 1M and base of cell 1Rs, veins largely black (Figure 8A); body hairs largely black, hairs on mesopleuron, abdominal sterna (Figure 8H), coxae and femora black largely except for yellowish brown basal 0.2.

**Sculptures.** Body weakly punctured and feebly microsculptured; head sparsely punctured, frontal area distinctly microsculptured; mesonotum sparsely, broadly and shallowly punctured, interspaces smooth shiny, mesopleuron deeply and densely punctured, weakly microsculptured; abdominal terga 1–2 sparsely and weakly punctured, both sides of median longitudinal ridge slightly microsculptured, other terga without punctures and microsculptures; middle and hind coxae and femora with sparse punctures and distinct microsculptures.

**Head.** Head with dense and long hairs, malar space 1.3× diameter of middle ocellus (Figure 8C); distance between inner margins of eyes as long as longest axis of eye; frontal area oval, front carina weak, concave in middle (Figure 8F); POL: OOL: OCL = 1: 0.9: 1.9 (Figure 8F); postocellar furrow thin and weak, middle furrow wide and shallow (Figure 8B); postocellar area 1.3× broader than long, anterior part of lateral furrow thin and shallow, posterior part indistinct, slightly divergent downwards; antenna with 7 antennomeres, antennomeres 6 and 7 with distinct segmentation.

**Thorax.** Middle of median mesoscutal groove slightly wide, anterior and posterior thin and weak, notaulus normal; anterior margin of mesoscutellum broadly arcuate; metascutellum blunt and straight; cenchri willow leaf shaped, distance between cenchri 2.4 × longest axis of a cenchrus; mesonotum hairs short and sparse (Figure 8D), mesopleuron hairs slightly dense and long (Figure 8G); vein 2m-cu joining cell 2Rs on outer side of vein 1r-m (Figure 8A), distance about 1/3 length of vein 1r-m, basal 1/3 of anal cell with a dot-like petiole, without crossvein (Figure 8L); middle and hind coxae and femora clearly elongated, ventral side of hind femur with two distinct longitudinal carinae, outer carina higher than inner one, with 1 distinct and large denticle near apex.

**Abdomen.** Middle of abdominal tergum hairs sparse and short (Figure 8I); subgenital plate developed, anterior and hind margins rounded (Figure 8M); apex lobe groove of penis valves broad, anterior margin arcuate, height more than width of sclerotized dorsal margin, posterior angle of apex lobe located front side of valvispina, protruding angle of paravalva located in middle of apex lobe (Figure 8N).

**Distribution:** China (Sichuan).

**Etymology:** The specific epithet is derived from the type locality.

#### 3.2.9. *Asitrichiosoma poecilomallosum* Yan & Wei, sp. nov.

urn:lsid:zoobank.org:act:E733DFA8-ADB6-40B7-881F-62D91CDB80D8

(Figure 9A–L)

**Material examined. *Holotype*** female, CHINA: Hubei Province, Mt. Shennongjia, Honghuaduo, 1200 m, 3 July 2007, leg. Meicai Wei (ASMN).

**Paratypes.** 1 female, CHINA: Chongqing Municipality, Shizhu County, Dafengbao, Huangshui, 3 May 2006, leg. Weiwei Zhang; 2 males, CHINA: Hunan Province, Wugang City, Mt. Yun Yunfengge; 1100 m, 26°38.963′ N, 110°37.169′ E; 25 April 2005; leg. Meicai Wei. 14 April 2013; leg. Zejian Li; 1 male, CHINA: Zhejiang Province Longquan City, Mt. Fengyang Shangxuqiao, 1500 m, 27.884° N, 119.174° E, 17 April 2023, leg. Zejian Li and Mingxuan Liu. 1 male, CHINA: Zhejiang Province, Qingyuan County, Baishanzu, 597 m, 27.540° N, 119.064°E, 7 April 2022; leg. Zhicheng Zhu (ASMN).

**Variation:** Some male specimens exhibit the absence of the vein 1r-m on the fore wing.

**Diagnosis:** The species is similar to *A. gansuense* Yan & Wei, 2025, but it differs from the latter in the following characters: median mesoscutal lobe, mesoscutellum and pleuron with yellowish brown hairs, head, lateral mesoscutal lobe, both sides of tergum 3 and most of tergum 4 with black hairs, other abdominal terga with yellowish brown hairs; antenna 1.4× head breadth (Figure 9F).

**Description.** Female. Body length 20.5 mm, black, medium to large size, thick and strong (Figure 9A).

**Coloration.** Apex of mandibles reddish brown (Figure 9E); abdominal terga 7–8, hind margins of sternum and abdominal 8–9 reddish brown; sheath black; wings yellowish and hyaline, infuscate macula covering cell 1M, pterostigma black, veins largely black, except for vein C and anal vein in fore wing reddish brown, veins C, Sc + R, and J in hind wing yellowish brown (Figure 9A); coxae, trochanters and fore femur black, middle and hind femora brownish black, tibiae and tarsus dark brown; head hairs largely black, gena, vertex and hind orbit mixed hairs black at base, yellowish brown at apex, pronotum, lateral mesoscutal lobe, both sides of tergum 3 and most of tergum 4 with black hairs, hairs on pleuron mesoscutellum, metanotum and abdominal tergum 1 yellowish brown, hairs on posterior margin of abdominal tergum 6 and terga 7–8 reddish brown; sheath with short and brown reddish hairs.

**Sculptures.** Body finely punctured, interspace between punctures shiny in head and thorax, postocular area and abdomen without luster.

**Head.** Malar space 1.3× diameter of middle ocellus; distance between inner margins of eyes 0.9× longest axis of eye; frontal area oval, median depression shallow (Figure 9E); frontal carina low and blunt, nearly straight, slightly convergent; POL: OOL: OCL = 4: 4.5: 5; postocellar area nearly square, postocellar furrow distinct, lateral furrow deep (Figure 9C); antenna with 7 antennomeres, 1.4× head breadth, antennomere 3 about 1.5× antennomeres 4 and 5 combined, club segmented distinctly, 1.3× antennomeres 4 and 5 combined, weak segmentation marks in the middle of antennomere 7 (Figure 9F).

**Thorax.** Distance between cenchri 1.8× longest axis of a cenchrus; metascutellum protruding; mesopleuron and scutellum with dense long hairs, median and lateral mesoscutal lobe with sparse hairs; metascutellum and metapleuron with slightly dense hairs; veins 1r-m and 2m-cu in fore wing completely joining at apex (Figure 9A).

**Abdomen.** Each abdominal tergum covered with symmetrical and dense hair; lancet with 49 serrulae (Figure 9I), membranous area between serrulae protruding, distance between serrulae about 1.6× basal breadth of a serrula, middle serrulae slightly protruding, subbasal teeth slightly large, each side with about 5 min subbasal teeth (Figure 9J).

**Male:** Body length 26–27 mm. Body hairs distinctly dense and long, with multicolored hair forming regular alternating transverse bands from head to the end of abdomen in dorsal view, pronotum, anterior part of mesonotum and anterior pleuron with yellowish white hairs, posterior part of mesonotum, mesepimeron and metanotum with black hairs (Figure 9B); mesoscutellum, metascutellum and abdominal terga 1–3 with yellowish white hair; both sides of tergum 3, terga 4 and 5 with black and short hair; posterior half of terga 5–7 with reddish brown hair; tergum 8 with golden-yellow hairs; sheath margins dense, short brown hairs.

**Distribution:** China (Hunan, Hubei, Chongqing, Zhejiang).

**Etymology:** The new species name is derived from the Latin words “poecilo” and “mallosum”, reflecting the species’ distinctive, attractive body hairs.

**Variation:** Some male specimens exhibit the absence of the vein 1r-m on the fore wing.

#### 3.2.10. *Asitrichiosoma shennong* Yan & Wei, sp. nov.

urn:lsid:zoobank.org:act:597063D9-E995-4490-83C6-2F0AF32DF819

(Figure 10A–K)

**Material examined. *Holotype*** female, CHINA: Hubei Province, Yichang City, Mt. Shennongjia Guitouwan, 2150m, 31°28.439′ N, 110°08.872′ E, 19 May 2012, leg. Ze-Jian Li (ASMN).

**Figure 10:**
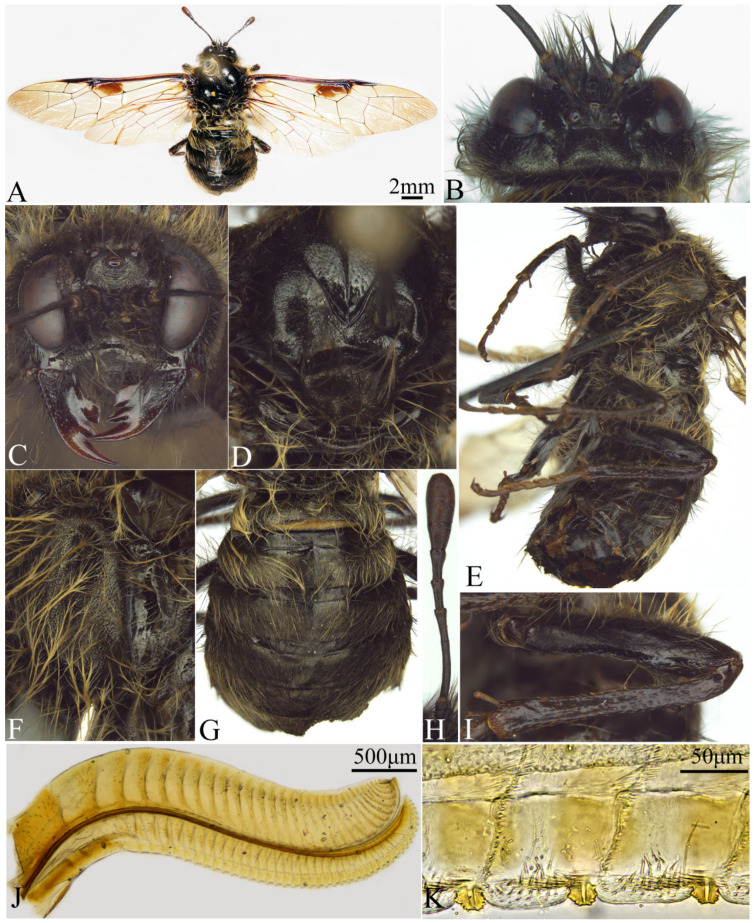
*Asitrichiosoma shennong* Yan & Wei, sp. nov. (**A**) Female adult, dorsal view; (**B**) head of female, dorsal view; (**C**) head of female, front view; (**D**) mesonotum; (**E**) female adult, lateral view; (**F**) mesopleuron and metapleuron; (**G**) abdominal terga; (**H**) antenna; (**I**) hind femur and tibia; (**J**) lancet; (**K**) middle serrulae.

**Diagnosis.** The species is similar to *A. sinense* Yan & Wei sp. nov., but it differs from the latter in the following characters: femora and tibiae entirely black (Figure 10I); abdominal terga 7–8 black; mesoscutellum and mesopleuron densely microsculptured, only finely punctured; veins 1r-m and 2m-cu in fore wing nearly joining at apex, veins M + Cu and cu-a nearly joining at apex (Figure 10A); membranous area between serrulae plat, distance between serrulae about 3.1× basal breadth of a serrula (Figure 10K).

**Description.** Female. Body length 14 mm (Figure 10A).

**Coloration.** Body black; wings pale yellow hyaline, pterostigma black, infuscate macula covering most of cell 1M, veins largely black (Figure 10A); tarsus yellowish brown; head hairs largely black, vertex mixed long hairs blackish brown at basal 2/3 and yellowish brown at apical 1/3, mesonotum largely covered with black hairs, mesoscutellum hairs blackish brown at basal 2/3 and yellowish brown at apical 1/3; mesopleuron and abdominal terga 1–2 with yellow hairs (Figure 10F), other terga and sterna largely covered with blackish brown hairs (Figure 10G), lateral margin hairs yellowish brown (Figure 10E); coxae and femora with yellowish brown hair (Figure 10I).

**Sculptures.** Body densely microsculptured, finely punctured; mandibles distinctly punctured, interspace between punctures smooth and shiny at apex; mesoscutellum and mesopleuron slightly densely punctured; abdomen terga without punctures, except for tergum 1.

**Head.** Clypeus thickened; malar space 1.4× median ocellus diameter; inner margins of slightly convergent downward, distance between below margins of eyes as long as longest axis of eye; frontal area circular, with low and blunt frontal carina (Figure 10C); POL: OOL: OCL = 2.5: 2.5: 3.3 (Figure 10B); postocellar furrow fine, middle furrow broad and shallow; postocellar area 1.7× broader than long, lateral furrow fine and shallow, slightly divergent backward; antenna with 7 antennomeres, 1.5× head breadth, antennal club segmented distinctly, 1.4× antennomeres 4 and 5 combined (Figure 10H).

**Thorax.** Median mesoscutal groove and notaulus distinct; anterior margin of mesoscutellum broad and arcuate; cenchri long and narrow, distance between cenchri 2.1× longest axis of a cenchrus; pronotum and mesonotum with sparse hairs, mesopleuron and metascutellum hairs dense and long; veins 1r-m and 2m-cu in fore wing nearly joining at apex, veins M + Cu and cu-a nearly joining at apex (Figure 10A).

**Abdomen.** Abdominal terga hairs slightly dense and long (Figure 10G); lancet with 40 serrulae (Figure 10J), membranous area between serrulae plat, distance between serrulae about 3.1× basal breadth of a serrula, middle serrulae not protruding, subbasal teeth indistinct, each side with about 3–4 min subbasal teeth (Figure 10K).

**Distribution:** China (Hubei).

**Etymology:** The specific epithet is derived from the type locality.

#### 3.2.11. *Asitrichiosoma sikkimense* (Konow, 1897)

*Trichiosoma sikkimense* Konow, 1897: 138-139.

urn:lsid:zoobank.org:act:F434AB05-B6B1-43C1-8AD8-AA035E45C1AD

(Figure 11A–N)

**Examined specimens:** 1 female, 1 male, Sikhim Coll. Bingham., det. Malaise 1939 (BMG).

**Figure 11:**
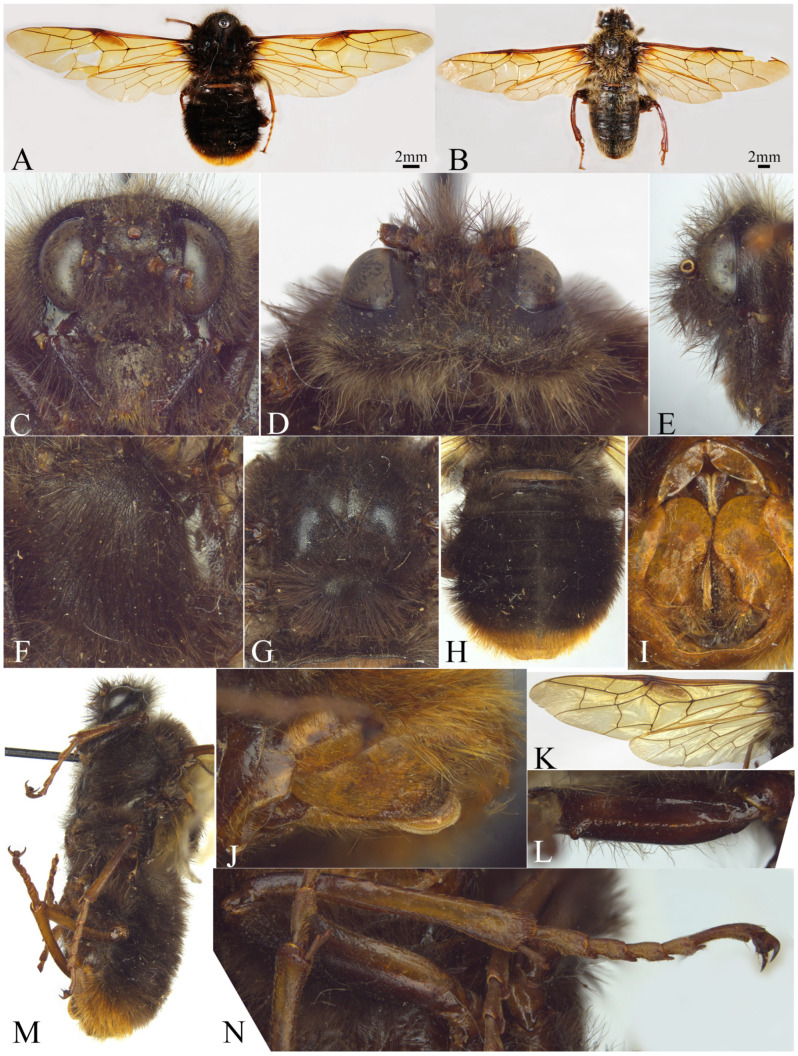
*Asitrichiosoma sikkimense* (Konow, 1897). (**A**) Female adult, dorsal view; (**B**) male adult, dorsal view; (**C**) head of female, front view; (**D**) head of female, dorsal view; (**E**) head of female, lateral view; (**F**) mesopleuron and metapleuron; (**G**) mesonotum; (**H**) abdominal terga; (**I**) ovipositor sheath, ventral view; (**M**) female adult, lateral view; (**J**) ovipositor sheath in lateral view; (**K**) wings; (**L**) hind femur; (**N**) hind leg.

**Diagnosis:** The species is similar to *A. nigropilosum* Yan & Wei sp. nov., but it differs from the latter in the following characters: the female abdominal terminal terga 1–2 with reddish brown hairs, remaining body hairs largely dark brown, male thorax and abdomen with light brown hairs; pterostigma in fore wing smoky macula cover the upper half of cell 1M (Figure 11A); median furrow of mesoscutal middle lobe normally well developed, not widened; vein 2m-cu in fore wing distinctly curved, vein cu-a joining the apex of vein 1M (Figure 11K); 1st abdominal tergum with weak microsculptures, surface distinct shiny; female femora and tibiae not entirely black (Figure 11L).

**Description.** Female. Body length 20 mm, black (Figure 11).

**Coloration.** Mandibles apically and ocellus brown (Figure 11C); abdominal terga 7–8 reddish brown, sterna dark brown, sheath reddish brown (Figure 11I); wings yellowish brown hyaline, fore wing brown macula covering upper part of cell 1M, pterostigma and wing veins black, vein C, Sc + R middle, M + Cu, anal vein in fore wing yellowish brown, vein C posterior, Sc + R, vein A and J in hind wing yellowish brown (Figure 11K); legs largely black, ventral side of hind coxae and femora dark brown, tibiae and tarsus yellowish brown; body with dense long hairs, except for reddish brown hairs on abdominal terga 7–8; edges of sheath with reddish brown short hairs, rest of body hairs black–brown.

**Sculptures.** Body minutely and densely punctured; head dim, terga and metapleuron interspace between punctures smooth, shiny; abdominal tergum 1 weakly punctured and microsculptured, slightly luster, other terga without luster; abdominal sterna, coxae and femora with strong and oily sheen.

**Head.** Labrum 0.5× basal breadth of clypeus, less than distance between the inner margins of eyes, anterior margin arcuate; clypeus 1.35× distance between lower margins of eyes, anterior margin with broad and arcuate depression, malar space 1.5× diameter of middle ocellus; distance between inner margins of eyes as long as longest axis of eye; frontal area subquadrate, shallow depression in middle; middle of frontal carina depression, nearly straight, postocellar furrow indistinct; POL: OOL: OCL = 2.3: 2.6: 4.5; postocular area slightly broader than long, lateral furrow deep and subparallel; postocular area slightly enlarged.

**Thorax.** Median mesoscutal groove normally well developed; cenchri ovate, distance between cenchri 2.2× longest axis of a cenchrus; metascutellum straight, not protruding; mesepisternum and scutellum with dense hairs, completely cover surface, median and lateral mesoscutal lobe slightly sparser hairs, metapleuron with slightly dense hairs; vein 2m-cu joining cell 2Rs on inner side of vein 1r-m, vein 2m-cu straight, vein cu-a directly joining vein 1M at apex, basal 1/3 of anal cell with a short crossvein; legs with strong oil sheen, dorsal side of middle and hind femora with dense long hairs, forming a brush-like structure.

**Abdomen.** Abdominal terga with dense long hairs.

**Male:** Body length 25 mm (Figure 11B), coloration and structure similar to the female, with the following exceptions: head and thorax largely brown; hairs on abdominal terga 1–2 dense, abdominal terga 3–6 hairs spare, abdominal terga 7–8 hair slightly dense.

**Distribution:** China (Himalayan region).

#### 3.2.12. *Asitrichiosoma sinense* Yan & Wei, sp. nov.

**Figure 12:**
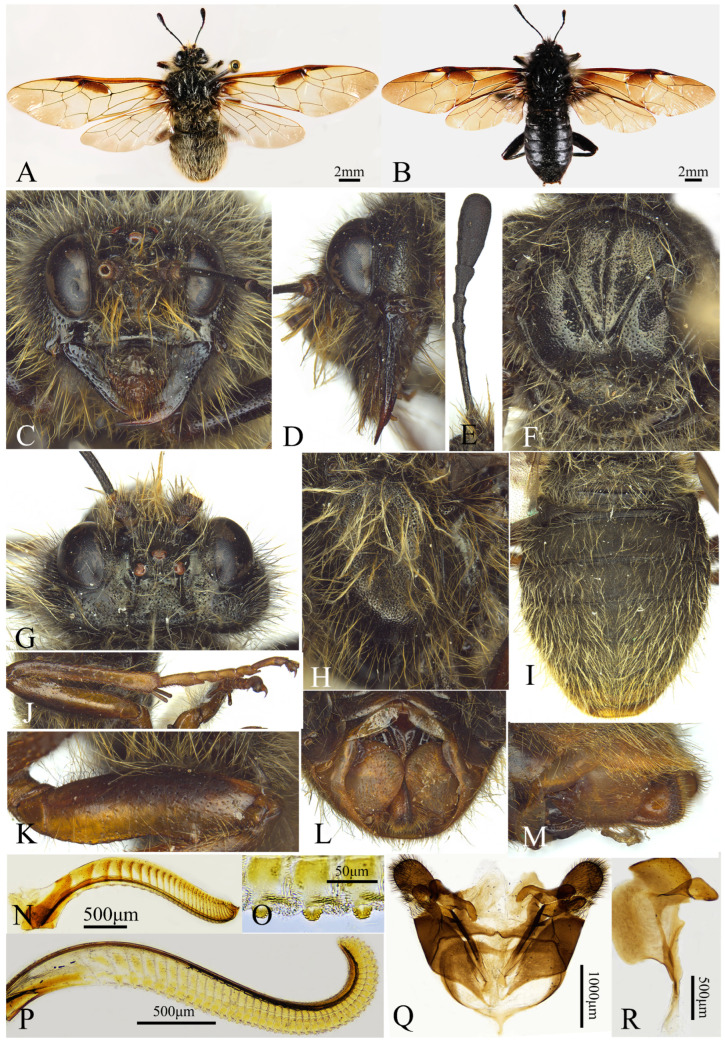
*Asitrichiosoma sinense* Yan & Wei, sp. nov. Female. (**A**) Female adult, dorsal view; (**B**) male adult, dorsal view; (**C**) head of female, front view; (**D**) head of female, lateral view; (**E**) antenna; (**F**) mesonotum; (**G**) head of female, dorsal view; (**H**) mesopleuron and metapleuron; (**I**) abdominal terga; (**J**) hind leg; (**K**) hind femur; (**L**) ovipositor sheath, ventral view; (**M**) ovipositor sheath, lateral view; (**N**) lance; (**O**) middle serrulae; (**P**) lancet; (**Q**) gonoforceps; (**R**) penis valve.

**Figure 13:**
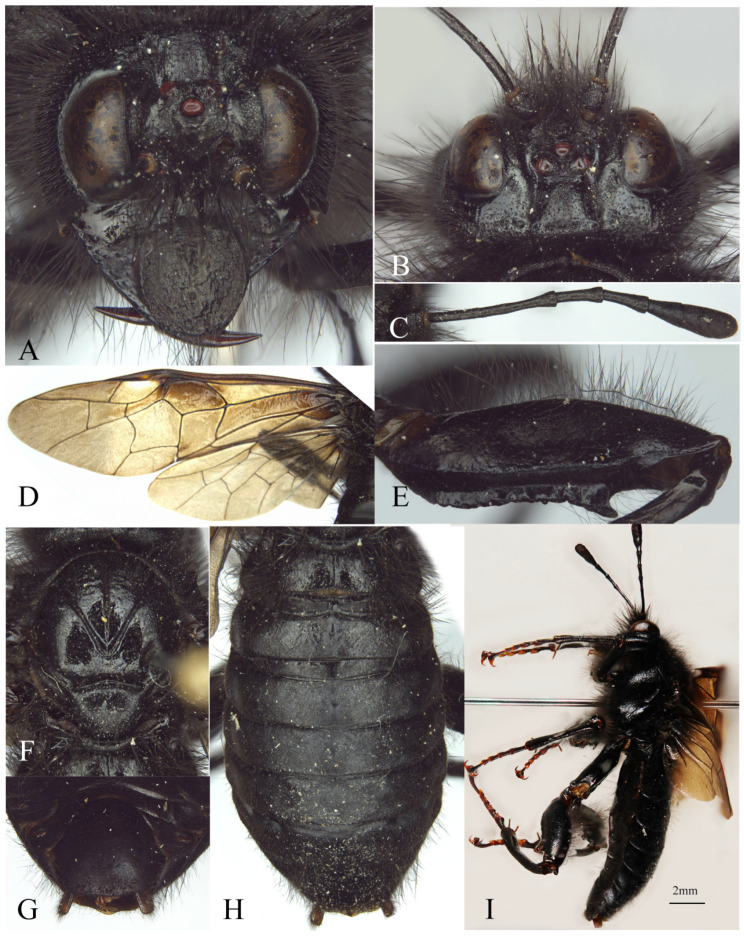
*Asitrichiosoma sinense* Yan & Wei, sp. nov. Male. (**A**) Head of male, front view; (**B**) head of male, dorsal view; (**C**) antenna; (**D**) wings of male; (**E**) hind femur; (**F**) mesonotum; (**G**) subgenital plate in ventral view; (**H**) abdominal terga; (**I**) male adult, lateral view.

urn:lsid:zoobank.org:act:82CB3D49-2F54-461E-AC36-574A9EFAD095

(Figure 12A–R and Figure 13A–I)

**Material examined. *Holotype*** female, CHINA: Sichuan Province, Mt. Emei, Gold Peak, 3067m, 29°31.369′ N, 130°20.188′ E; 3 July 2006, leg. Yihai Zhong (ASMN).

**Paratypes.** 3 males, CHINA: Sichuan Province; Mt. Emei, Gold Peak; 3077 m, 29°52.420′ N 103°34.030′ E; 13 June 2007, leg. Yihai Zhong, Shaobing Zhang (ASMN).

**Diagnosis.** The species is similar to *A. shennong* sp. nov., but it differs from the latter in the following characters: ventral side of femora and tibiae reddish brown (Figure 12J,K); female hind margins of abdominal terga 7–8 pale brown (Figure 12L); mesoscutellum and mesopleuron deeply and distinctly punctured; veins 1r-m and 2m-cu in fore wing completely joining at apex, veins M + Cu and cu-a joining at apex (Figure 12A); membranous area between serrulae protruding, distance between serrulae about 1.4× basal breadth of a serrula (Figure 12O).

**Description.** Female. Body length 14 mm, body black (Figure 12A).

**Coloration.** Labrum reddish brown, cenchrus blackish brown; wings largely brown and hyaline, fore wing veins black, except for anal cell veins yellowish brown, pterostigma black, infuscate macula covering 2/3 cell 1M and base of cell 1Rs, hind wing veins largely black, veins Sc + R, M yellowish brown (Figure 12A); legs pale brown, dorsal part of coxae (Figure 12J), middle and hind femora with black longitudinal stripes (Figure 12K); hairs on vertex and gena yellowish brown largely except for black basal 0.2, hairs on hind orbit blackish gray at base and yellowish white at apex (Figure 12C), hairs on pronotum, mesonotum yellowish white largely except for black basal 0.2, hairs on mesopleuron, abdominal terga 1–7, sterna and coxae and femora yellowish white, tergum 8 and sheath hairs brown, sheath with black setae.

**Sculptures.** Head distinctly microsculptured, postocular area finely punctured and slightly shiny; labrum and outer side of mandible with broad and shallow punctures, interspaces smooth, shiny; pronotum and mesopleuron deeply and distinctly punctured, mesosternum broadly and sparsely punctured; abdominal tergum 1 clearly and densely punctured and microsculptured, other terga without punctures but densely microsculptured, abdominal sterna broadly and sparsely punctured, interspaces smooth; middle and hind coxae and femora broadly and sparsely punctured, interspaces smooth, shiny.

**Head.** Clypeus and labrum thickened in middle; malar space 1.5× diameter of middle ocellus, distance between inner margins of eyes 1.1× longer than longest axis of eye; frontal area subquadrate, with a distinct frontal carina; median fovea shallower than lateral fovea; POL: OOL: OCL = 3: 3.3: 5.4 (Figure 12G); postocellar furrow feeble, middle furrow distinct; postocellar area 1.4× broader than long, lateral furrow fine and deep, anteriorly straight and parallel, posteriorly curved and slightly divergent; antenna with 7 antennomeres, 1.6× head breadth, antennomere 3 about 1.7× antennomeres 4 and 5 combined, antennal club segmented, 1.3× antennomeres 4 and 5 combined (Figure 12E).

**Thorax.** Median mesoscutal groove and notaulus clear, anterior margin of mesoscutellum shallow arc shaped, metascutellum blunt, not protruding; cenchri long-oval, distance between cenchri 1.75× longest axis of a cenchrus; pronotum and mesonotum with sparse long hairs, mesopleuron with dense, long hairs, mesoscutellum and metascutellum with dense and long hairs; veins 1r-m and 2m-cu in fore wing completely joining at apex, veins M + Cu and cu-a completely joining at apex (Figure 12A,); coxae and femora with sparse and long hairs, inner margin of fore tibia with dense, short hairs (Figure 12K).

**Abdomen.** Abdominal terga with dense and long hairs, abdominal terga 1–5 with dense hairs, apex sparse (Figure 12I); lancet with 45 serrulae, membranous area between serrulae protruding, distance between serrulae about 1.4× basal breadth of a serrula, middle serrulae protruding, subbasal teeth slightly large, each side with about 5 min subbasal teeth (Figure 12O).

**Male:** Body length 13.5–17 mm, body and body hair entirely black (Figure 13I); mandibles apex and ocellus reddish brown (Figure 13A); wings dark smoky brown (Figure 13D); tarsal pulvillus and claws reddish brown; head and thorax with dense and long hairs, densely microsculptured, scattered and sparsely punctured; abdominal terga with sparse hairs (Figure 13H).

**Distribution:** China (Sichuan).

**Etymology:** The specific name of the new species refers to the distribution area, China.

### 3.3. Distribution of Asitrichiosoma

**Figure 14:**
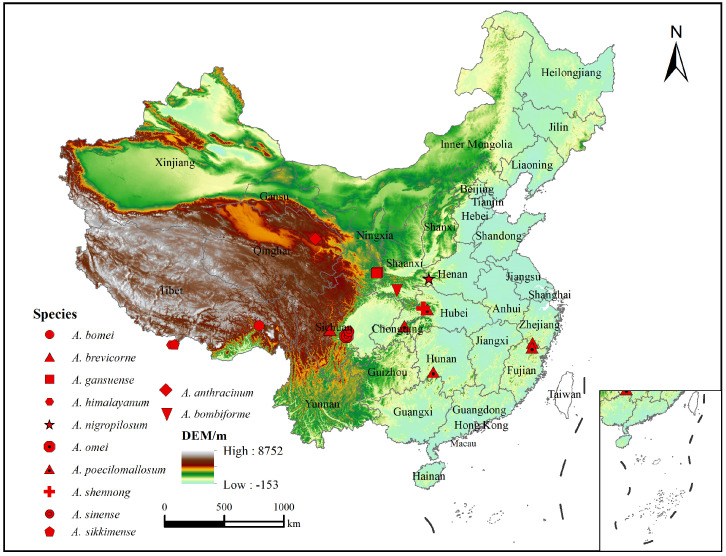
Distribution of the examined species within *Asitrichiosoma*.

The examination of specimen records for the genus *Asitrichiosoma* reveals its distributional characteristics (Figure 14). The genus is reported from East Asia, and the records of its distribution spans eastern and western China, extending from southwest Tibet to the coastal region of East China (Zhejiang), encompassing multiple provincial-level administrative divisions (Tibet, Qinghai, Sichuan, Shaanxi, Gansu, Henan, Hubei, Chongqing, Hunan, and Zhejiang), as well as historical records from Sikkim (now part of India). Several narrow-range endemic species are recognized, including *A. bomei* (Bomi, Tibet), *A. omei* (Mount Emei, Sichuan), and *A. shennong* (Shennongjia, Hubei). By contrast, *A. poecilomallosum* exhibits the broadest distribution, occurring across five provinces and municipalities.

#### The Worldwide Species List of *Asitrichiosoma*

1. *A. anthracinum* (Forsius, 1930) comb. nov.
  Distribution: China (Northeastern, Qinghai)
2. *A. bombiforme* (Takeuchi, 1939) comb. nov.
  Distribution: China (Gansu, Shaanxi), Japan
3. *A. bomei* Yan & Wei, sp. nov.
  Distribution: China (Tibet)
4. *A. brevicorne* Yan & Wei, sp. nov.
  Distribution: China (Sichuan)
5. *A. gansuense* Yan & Wei, 2025.
  Distribution: China (Gansu)
6. *A. himalayanum* (Malaise, 1939) comb. nov.
  Distribution: China (Himalayan region)
7. *A. nigropilosum* Yan & Wei, sp. nov.
  Distribution: China (Henan, Shaanxi)
8. *A. omei* Yan & Wei, sp. nov.
  Distribution: China (Sichuan)
9. *A. poecilomallosum* Yan & Wei, sp. nov.
  Distribution: China (Hubei, Hunan, Chongqing, Zhejiang, Sichuang)
10. *A. shennong* Yan & Wei, sp. nov.
  Distribution: China (Hubei)
11. *A. sikkimense* (Konow, 1897) comb. nov.
  Distribution: Himalayan region
12. *A. sinense* Yan & Wei, sp. nov.
  Distribution: China (Sichuan)

## 4. Discussion

Historically, *Asitrichiosoma* was established as a subgenus of *Trichiosoma* by Malaise (1939) based on certain overall morphological similarities between the two groups. However, a comprehensive morphological comparison among *A. himalayanum*; *A. sikkimense*; *A. gansuense* Yan & Wei, 2025; the seven new species described herein; and the *T. vitellinae* of the genus *Trichiosoma* reveals that the newly elevated genus exhibits stable and significant differences from *Trichiosoma* in several key diagnostic morphological traits. These differences include head structure, wing venation, leg architecture, and pubescence patterns. The male genitalia, particularly the structure of the penis valve, together with the leg configurations represent critical diagnostic characters in the Cimbicidae taxonomy. In *Asitrichiosoma*, these structures display consistent and conspicuous differentiation from those observed in *Trichiosoma*, further supporting the taxonomic treatment of establishing it as a distinct genus.

In the phylogenetic tree, both maximum likelihood and Bayesian analyses recovered *Asitrichiosoma* and *Trichiosoma* as sister groups, indicating a close phylogenetic relationship between them. However, *A. poecilomallosum* and *A. anthracinum* form a clade that is clearly separated from *Trichiosoma*, supporting the distinctness of *Asitrichiosoma*. Genetic distance analysis revealed an intrageneric distance of 0.0256 within *Trichiosoma*, whereas the intergeneric distance between *Asitrichiosoma* and *Trichiosoma* was 0.0878. Our statistical analyses demonstrated significant differences between the two genera, indicating that *Asitrichiosoma* is distinct from *Trichiosoma*. However, because of the small sample size of *Asitrichiosoma* in this study, the molecular evidence merely lends tentative support to the morphological data.

Wang et al. (1992) [33] reported two pest species of Cimbicidae, namely, *T. bombiforme* and *T. anthracinum*. Of these, *T. bombiforme* is herein transferred to the genus *Asitrichiosoma*. However, a comparison of the morphological characteristics of the reported *T. anthracinum* revealed that the specimen identified as *T. anthracinum* by Wang (1992) differs markedly from the original description of Forsius (1930). Forsius (1930) described *T. anthracinum* as having an entirely black abdomen covered with relatively dense blackish grey pubescence. In contrast, the specimens described by Wang. (1992) have the entire thorax and abdominal tergites 1–4 covered with black pubescence, with only tergites 5–8 covered with brown pubescence, which does not conform to the original description of Forsius (1930). Based on these morphological differences, we consider that the specimen reported as *T. anthracinum* by Wang (1992) does not represent the species described by Forsius (1930), but instead belongs to the genus *Asitrichiosoma*. According to the morphological characteristics reported for the *T. anthracinum*, it should be attributed to *A. nigropilosum* Yan & Wei, sp. nov. established in the present study.

The genus *Asitrichiosoma* is predominantly distributed in the mountainous regions of central and southwestern China, with records spanning approximately 26° N to 34° N in latitude. Most species occur at elevations above 1000 m, indicating a strong association with montane forest ecosystems. Sichuan Province represents the current known center of diversity, harboring three species, including two sympatric species (*A. omei* and *A. sinense*) on Mt. Emei. This concentration likely reflects the topographic complexity and habitat heterogeneity characteristic of the Hengduan Mountains region. *A. poecilomallosum* exhibits the widest geographic range within the genus, occurring across the Daba, Wuling, and Wuyi Mountains, suggesting considerable ecological tolerance.

Based on detailed morphological evidence, we propose the removal of *A. anthracinum*, its allied species, and the seven new species described herein from the genus *Trichiosoma*, and the elevation of *Asitrichiosoma* from subgeneric to generic rank. This taxonomic treatment is founded upon consistent and significant morphological discontinuities in multiple character systems, including male genitalia, wing venation, and pubescence patterns. Molecular data for *Asitrichiosoma* are currently insufficient; future studies incorporating molecular evidence will further test the taxonomic hypothesis presented in this study.

## Data Availability

The data presented in this study are available in this article.
